# Kinetic and isotherm study of Ni-MOF/Magnetite nanoparticles adsorption capacity as green synthesized adsorbent towards rhodochrome (Kammererite)

**DOI:** 10.1038/s41598-025-29707-7

**Published:** 2025-12-28

**Authors:** Mostafa F. Elshafei, Maysa R. Mostafa, Perihan A. Khalf-Alla, Gehad G. Mohamed, Omar A. Fouad

**Affiliations:** 1 Department of Chemistry, Faculty of Science, Cairo University, Giza, 12613 Egypt; 2 Department of Nanoscience, Faculty of Basic and Applied Sciences, Egypt-Japan University of Science and Technology, New Borg El Arab, Alexandria, 21934 Egypt

**Keywords:** Ni-MOF/Magnetite NPs, Rhodochrome, Kammererite, Tea extract, Adsorption, Isotherms, Kinetics, Chemistry, Environmental sciences, Materials science, Nanoscience and technology

## Abstract

This study presents a novel approach by batch adsorption method for the removal of Rhodochrome (Kammererite) from wastewater via a sustainable, environmentally friendly, natural-dependent synthesis to produce Ni/MOF/tea-magnetite nanoparticles as adsorbent. No approach for the elimination of this category of chlorite was discussed using the adsorption batch method, which is highly efficient in relation to cost and time, as well as being rapid and environmentally benign. The study will also consider pH, nanoparticle dose, dye concentration, agitation speed, temperature, and contact time to evaluate how this influence removal efficiency, as well as isotherms, kinetics, and nanoparticle reusability as a cost-saving strategy. The morphology and microstructure of the adsorbent material were investigated using scanning electron microscopy (SEM), transmission electron microscopy (TEM), Brunauer-Emmett-Teller (BET) surface area analysis, zeta potential measurement, and X-ray diffraction (XRD).The optimum removal parameters were pH 8, a duration of 5 min at 600 rpm, and 0.07 g of nanoparticles, resulting in a clearance rate of 93%. Moreover, it was shown that the material may be utilized for three cycles with a clearing rate of 85%. The adsorption process can be conducted without modifying the temperature. The adsorption isotherm and kinetics studied was evaluated using the different kinetics study, Langmuir, Freundlich, DKR, and Temkin models. The removal procedure was determined to comply with the Temkin model and pseudo second order reaction.

**Supplementary Information:**

The online version contains supplementary material available at 10.1038/s41598-025-29707-7.

## Introduction

Water is frequently contaminated with pollutants such as heavy metals, inorganic chemicals, and organic dyes, which can endanger human health and cause a variety of diseases^1–4^. However, about 80% of the wastewater contains dyes that are released untreated into rivers or applied directly to farming^5^. Organic dyes are thought to be serious water pollutants. Dye exposure has been linked to severe health risks for humans and other organisms. It may cause liver, brain, and nervous system, renal and reproductive dysfunction^6^. Given the rising demand for water and the contamination of the environment, this poses a threat not only to humans but to all living things^7^. Especially, the basic and diazo direct dyes showed the highest rates of toxicity. Wastewater treatment has shifted its focus to the printing and dyeing industries, which use dyes for coloring^8^. Various kinds of pollutants may exist in significant quantities within industrial wastewater.

Specific organic and inorganic hazardous chemicals, including complex dyes and heavy metals, are derived from the mining and galvanizing sectors. These pollutants disturb the ecosystem and organisms, leading to various serious diseases. Therefore, the development of alternative technologies must be efficient, adequate, economical, and environmentally friendly^9–11^.

Chromian clinochlore, or kämmererite, is stunning, featuring vibrant pink, purple, rose, and scarlet colors. Kämmererite is micaceous and has perfect basal cleavage, making it difficult to facet. Faceted gems are rare and tiny. The predominant members of the chlorite group which consists of prevalent phyllosilicates containing magnesium, iron, and aluminum comprise: clinochlore (magnesium-rich), chamosite (iron-rich), pennantite (manganese-rich), and nimite (nickel-rich)^12–18^. The clinochlore variations utilized as semi-precious gemstones include seraphinite, kammererite, and sheridanite. Kammererite serves as an astrological talisman for the zodiac signs Virgo and Pisces. Kammererite is located alongside clinochlore and uvarovite in chromite deposits. Kammererite is primarily located and abundantly produced in the eastern Anatolia region of Turkey and many other places^12,13,19^.

It has formula [Mg_5_(Al, Cr, Fe)_2_Si_3_O_10_(OH)_8_], is a hydrous silicate exhibiting a monoclinic IIb-2 polytype and is exceedingly rare and much sought after by mineral collectors. Important group of the phyllosilicates or sheet silicate family of minerals, which are distinguished by layered structures, composed of polymeric sheets of SiO_4_ tetrahedral. Linked to sheets of (Al, Mg, Fe)(O, OH)_6_ octahedral. Kammererite was treated with epoxy to enhance its longevity due to its poor hardness. Furthermore, it has been established that they are suitable for usage in both jewelry and decorative items with the employed cabochon cutting techniques^19^.

According to the layer type, the magnitude of the net layer charge, the type of interlayer material, the character of the octahedral sheet, in addition to the composition or structure of individual species the net charge depending as shown in Supplementary Fig. 1. Which explains the 1:2-layer silicates? The chlorite group members contain a 2:1 layer with variable x and an interlayer hydroxide sheet. In some references, they are referred to as 2:1:1 mineral. The octahedral sheets may both be dioctahedral (di/di) or trioctahedral (tri/tri), or mixed (di/tri, or tri/di). The interlayer hydroxide sheet may have a positive charge. The pH of the environment plays a crucial role in determining the toxicity of a clay, as it can influence the release of potentially toxic metals and metalloids from the clay into the surrounding water^20^. Natural impurities present in the clinochlore mineral lattice, such as transition metals like chromium and manganese, can significantly affect its properties and potential toxicity^21^.

The ecotoxicity of clinochlore is not stable and can change, making it crucial to re-evaluate toxicity based on the specific mineralogical and chemical composition of the clay. They are used directly and indirectly in various cosmetic formulations to improve organoleptic and physicochemical properties and to increase stability^22^.

In the case of chlorites, which have two octahedral sheets as rhodochrome, the brucite sheet in the interlayer tends to be thinner than that in the 2:1 talc part because the former contains sufficient R^3+^ cations to sustain its positive charge^23^.

Samples containing above 2% Cr_2_O_3_ are designated as kotschubeite when chromium occupies tetrahedral sites and kemmererite when it resides in octahedral sites with trivalent oxidation state^24^.

Water–rock/soil interaction and humans control groundwater quality. Groundwater geochemistry is affected by rock/soil precipitation/dissolution, sorption, and others affect water quality alongside natural processes. Kammererite quickly dissolves in water when crushed into powder or minute particles, potentially releasing significant quantities of hazardous metals, particularly chromium and others^25,26^. So, a variety of methods to remediate wastewater, such as precipitation^27–29^, membrane filtration^30^, ion exchange^31^, biodegradation^32^ and Adsorption^33–37^ were studied. However, many methods produce hazardous sludges, are expensive to operate and maintain, and are complicated to remediate. Adsorption technique is quick, easy, cost-effective, and environmentally benign, making it extremely efficient technology. It reduces organic dyes and purifies wastewater^3,38–45^. Green chemistry emphasizes low costs, simple methods, no hazardous ingredients, and better process efficiency.

Nonetheless, no approach was proposed for the elimination of chromium-rich compounds, such as Chromium Clinochlore. Only a limited number of papers have been previously published on this substance, centering on spectroscopic studies^46–48^.

Recent advances in wastewater treatment have greatly benefited from nanoparticles’ unique properties, such as increased reactivity, adsorption capacities, and improved catalysis^49–52^.

Nanoscale particles have a wide range of unique optical, magnetic, and electrical properties, as well as a large surface area, higher surface energy, and quantum confinement^53,54^. Due to the elevated levels of polyphenols and caffeine, tea extract functions as the ideal reductant and stabilizer for the green synthesis of iron oxide NPs^55–57^. Magnetite Fe_3_O_4_ NPs are appealing for use in a variety of biomedical and catalytic applications due to their stability, biocompatibility, biodegradability, ease of surface modification, and narrow particle size distribution. A tea extract was employed to develop a biocompatible covering for magnetite nanoparticles^58–60^.

Metal-organic frameworks (MOFs) are intriguing porous materials composed of inorganic constituents that are linked by organic interconnections. Their versatility, ease of synthesis, structural design, and high surface area has attracted attention. Organic linkers and transition metal carboxylate clusters interact efficiently to generate extensive frameworks with exceptional stability. Their networks and simple counter ions serve as secondary building blocks for MOF synthesis^61–65^. This study will cover the removal of rhodochrome as an example of chlorite removal using the adsorption batch method, which is highly efficient in relation to cost and time, as well as being rapid and environmentally benign. An eco-friendly, room temperature method was used to synthesize Ni-MOF/Magnetite NPs as an adsorbent for removing rhodochrome from wastewater. The nanoparticles were characterized using XRD, SEM, TEM, zero-point charge, and BET. In along with isotherms, kinetics, and nanoparticle reusability as a cost-saving approach, removal efficiency metrics will be studied.

## Experimental

### Materials and methods

Terephthalic acid (Benzene-1,4-dicarboxylic acid) with a purity of 98% and Ni(III) acetate dihydrate with a purity of 97% and Ferric chloride hexahydrate with a purity of 97% and Iron(II) chloride tetrahydrate (purity of 98%) and NaOH (purity of 98%) were obtained from Sigma-Aldrich Chemie GmbH, located in Eschenstrasse, Germany. All glassware and equipment were cleaned with deionized water, which was utilized in all preparations.

### Instrumentation

The scanning electron microscope (SEM) image of the metal-organic framework (MOF) was acquired using the Quanta FEG250 SEM at the National Research Centre in Egypt. X-ray diffraction (XRD) was performed at the Egypt Nanotechnology Centre (EGNC) using a Bruker D8 Discover X-ray diffractometer (Bruker AXS Inc., 35 kV, 30 mA). The research utilized a step size of 0.02 and a scan speed of 0.016, employing Cu Kα radiation (λ = 1.5406 Å) over a length of 2 h, with 2θ values spanning from 5 to 50. Gas adsorption studies employing N2 as the adsorptive medium at 77 K have been conducted to determine BET surface area and pore size distribution. The samples were evacuated for 4 to 12 h prior to conducting the adsorption tests under high vacuum conditions. The analysis employed a Nova Touch LX2 analyzer, with calculations based on the Brunauer-Emmett-Teller (BET) theory.

### Experimental part

#### Extraction and determination of tea polyphenols

A typical tea bag was submerged in 100 milliliters of double-distilled water and allowed to boil for 30 min. To get a clear extract, the tea bag was taken out of the solution and filtered twice using filter paper. After that, the green tea extract was kept for further use at 4 °C^66^.

#### Synthesis of Fe_3_O_4_ by black tea extract

Aqueous solutions of Ferric chloride hexahydrate and Iron(II) chloride tetrahydrate were combined in suitable volume ratios to achieve a 2:1 ratio of Fe^3+^ to Fe^2+^ ions, after which green tea extract was incrementally added until a greenish-black solution was attained. The pH was incrementally raised to 10 by the dropwise addition of ammonia to the solution while maintaining continuous stirring at 600 rpm. The produced samples were rinsed with deionized water 4–5 times and subsequently dried by heating them in an oven at 60 °C for 12 h^66,67^.

#### Synthesis of nickel-metal-organic framework (Ni-BDC-MOF) by room temperature method

##### Firstly, Preparation of a 50 mL solution of 0.2 M sodium terephthalate (Na_2_TPA)

0.79 g of NaOH was dissolved in 20 ml of deionized water, after which 1.66 g of terephthalic acid (TPA) was added to the NaOH solution. The stirring was sustained at room temperature until the solution achieved clarity and transparency. The synthesis solution was then transferred to a 50 ml volumetric flask for volume calibration to get a 0.2 M Na_2_TPA solution^68^.

##### Secondary, Preparation of a 50 mL solution of 0.2 M nickel acetate

2.45 g of nickel acetate was dissolved in 30 ml of deionized water and then transferred to a 50 ml volumetric flask to prepare a 0.2 M nickel acetate solution.

##### Finally, Preparation of nickel terephthalic MOF (Ni-BDC-MOF) by room temperature method

The prepared 0.2 M nickel acetate solution was incrementally added to the 0.2 M Na_2_TPA solution dropwise under vigorous stirring, and stirring was maintained for 4 h. The solution was subjected to centrifugation, and the precipitate was washed with water three times, followed by three washes with ethanol to isolate the product. The product (Ni-BDC-MOF) was immersed in 50 ml of ethanol for 24 h, subsequently retrieved through centrifugation, dried for 12 h at 120 °C, and allowed to cool in ambient air for 20 min^68^.

#### Preparation of Ni-MOF \magnetite nanocomposite

Ni-MOF was incorporated into magnetite nanoparticles at a weight ratio of 1:1, followed by the addition of 20 ml of ethanol. The mixture was sonicated for 20 min, then agitated for 8 h at room temperature (25 °C), and subsequently dried at room temperature to yield Ni-MOF/magnetite nanocomposites.

### Adsorption studies

Adsorption experiments were conducted at a constant temperature of (25.0 ± 1.0 °C) using the adsorbent and 100 mL of dye solution. The pH of the assessed solutions was modified using 0.1 M NaOH or HCl solutions. The solution was agitated at a uniform pace in a 250 mL conical flask to perform the experiments. The analysis was performed thrice, and the adsorbent was isolated from all samples utilizing a 45 μm polyethylene membrane filter following the specified duration. The concentration of rhodochrome in samples collected at designated time intervals was quantified using a UV-Vis spectrophotometer (UVmini-1240, Shimadzu) at a wavelength of 530 nm. A multitude of variables were analyzed, encompassing pH value, adsorbent dosage, beginning dye concentration, contact duration, and shaking speed^3,45^. The subsequent Eq. (3) was employed to determine the percentage of dye removal:

1 $$Percentage{\text{ }}Removal{\text{ }} = {\text{ }}\left[ {\left( {C_{0} - {\text{ }}C_{e} } \right)/C_{0} } \right]{\text{ }} \times {\text{ }}100$$

C_0_ represents the initial dye concentration (mg/L), whereas C_e_ denotes the dye concentration after adsorption (mg/L). Additionally, the adsorption capacity of the adsorbent, q_e_ (mg dye per g of dry adsorbent), can be determined using the following Eq. (2):

2 $$q_{{\max }} = {\text{ }}\left( {C_{o} - {\text{ }}C_{e} } \right){\text{ }} \times {\text{ }}\left( {v/w} \right)$$

where V (in liters) represents the volume of the solution and w (in grams) denotes the quantity of dry adsorbent.

## Results and discussion

### Characterization of the sorbent

#### X-ray diffraction (XRD)

**Fig. 1:**
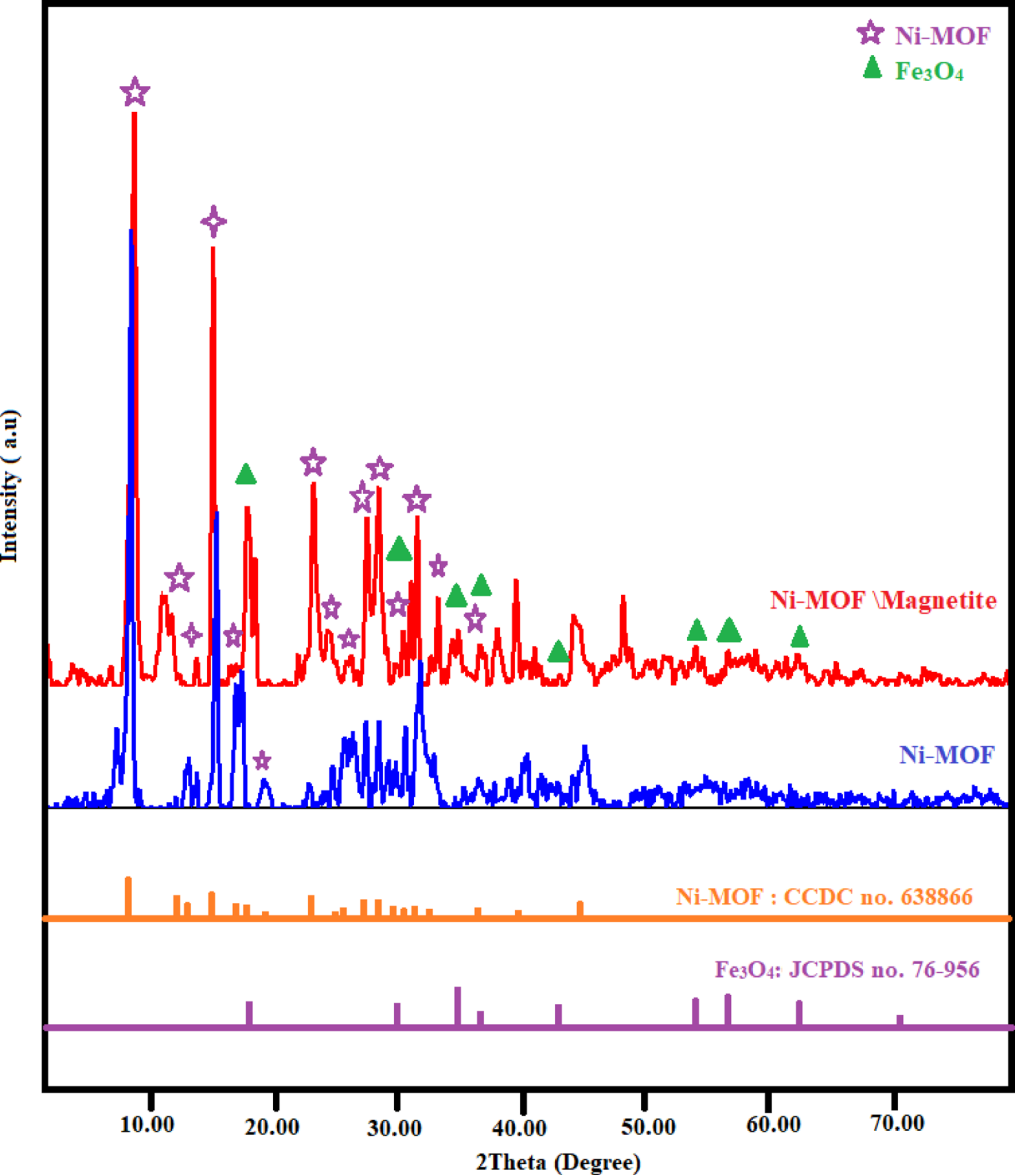
XRD pattern the nano Ni-BDC-MOF and Ni-MOF/magnetite.

The crystal phases of the synthesized Ni-BDC-MOF were examined through XRD analysis, with the results presented in Fig. 1. The XRD pattern showed sharp and intense diffraction peaks, which suggested the sample has high purity and a clear crystal phase. The XRD pattern of Ni-MOF aligned well with the previously prepared Ni-BDC-MOF^69,70^. The observed peaks exhibited a strong correlation probably had a layered topology of [Ni_3_(OH)_2_(C_8_H_4_O_4_)_2_(H_2_O)_4_].2H_2_O with a card number (CCDC no. 638866), characterized by the space group (*P*−1)) and a triclinic crystal structure^71^. This data demonstrated outstanding agreement of the synthesized Ni-BDC-MOF with the standard XRD pattern^69–71^. Table 1 presented an overview of the details related to the synthesized Ni-BDC-MOF crystallographic MOF^72,73^ and by comparison the XRD pattern of the synthesized Ni-BDC \magnetite with the XRD of Ni-MOF and the standard XRD of magnetite (card number JCPDS: 76–956), it was showed that a sharp and intense diffraction peaks of the synthesized Ni-MOF combined with the diffraction peaks of the synthesized magnetite nanoparticles. This result confirmed the successful synthesized of the Ni-BDC \magnetite nanocomposite. The Debye-Scherrer equation was also used to analyze the average crystallite size from the XRD patterns, and the results showed that the nanometric dimension of the Ni-BDC-MOF and Ni-BDC magnetite nanocomposite which also indicated by the prominent sharp peaks of the XRD pattern. Additionally, nearly all inorganic mesoporous materials fall within the 2θ range of 6–26^°^. Therefore, the mesoporous nature of the Ni-BDC magnetite nanocomposite was confirmed^74^.

**Table 1:** Crystallographic data of the synthesized Ni-BDC-MOF.

Crystal system	Triclinic
Space group	*P*−1
a (°A)	10.2077
b (°A)	8.0135
c (°A)	6.3337
Angle beta (*α)*	97.701 ^o^
Angle beta (*β)*	97.213 ^o^
Angle beta (*γ)*	108.767^o^
Z	1
I/Ic	1.610
Average crystallite size	24.9 nm

#### Scanning electron microscope (SEM)

**Fig. 2:**
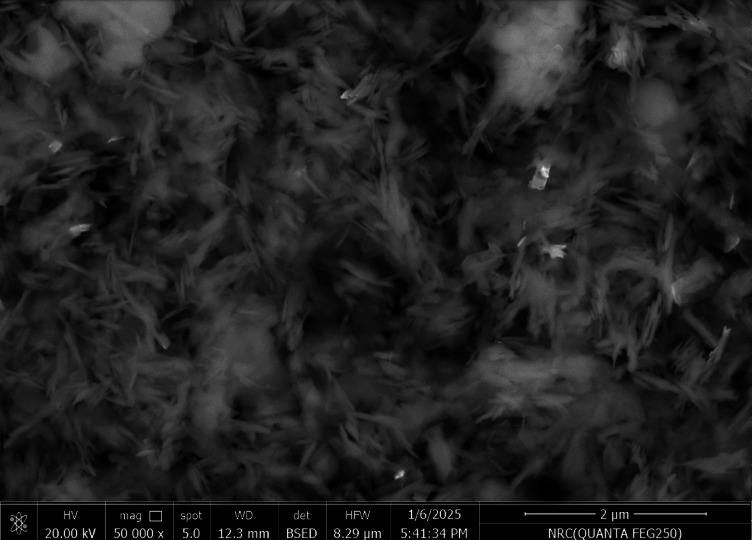

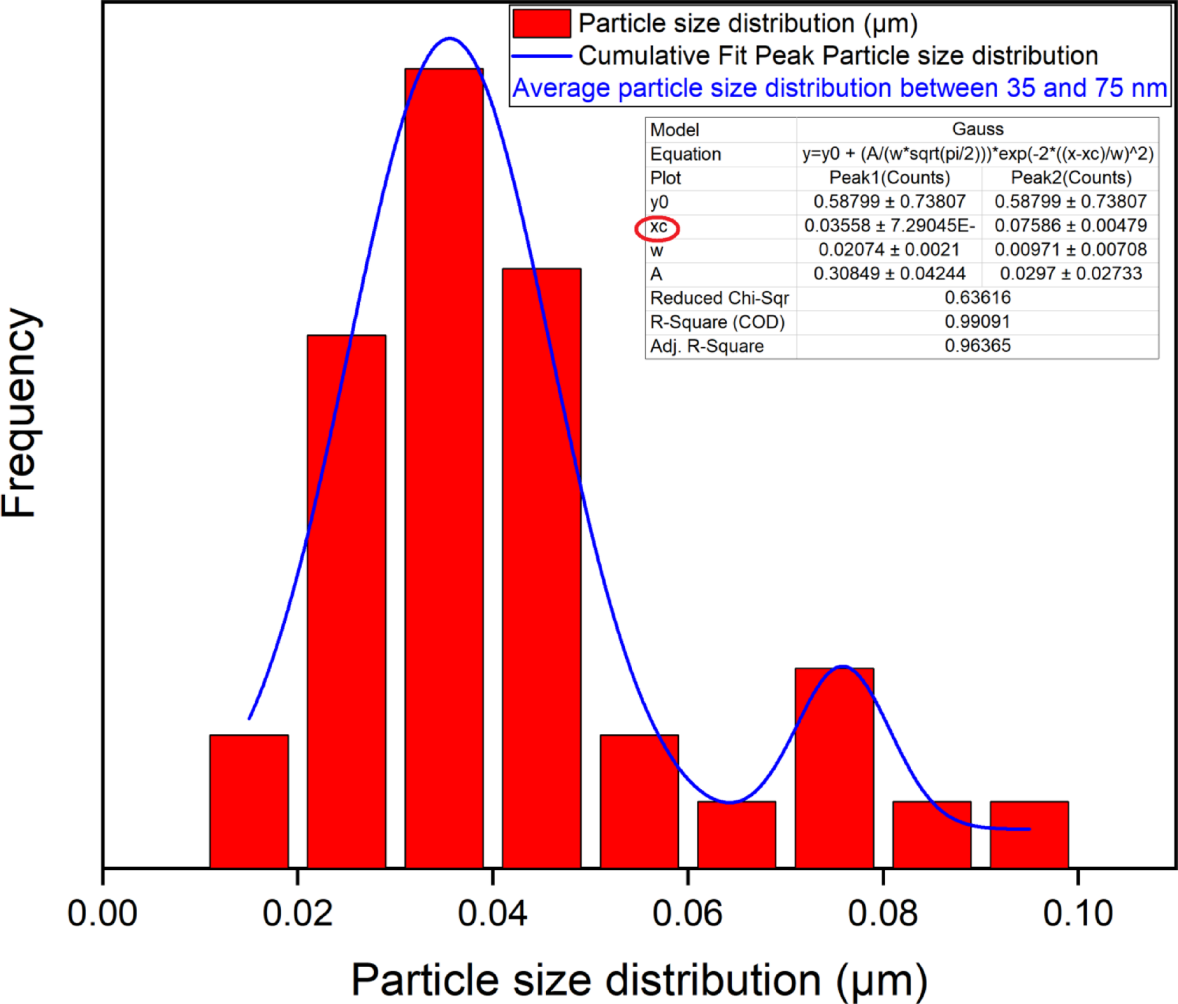

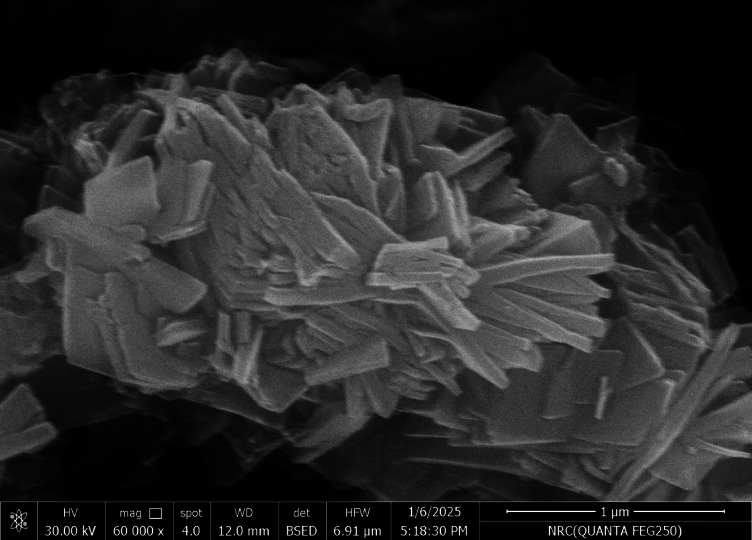

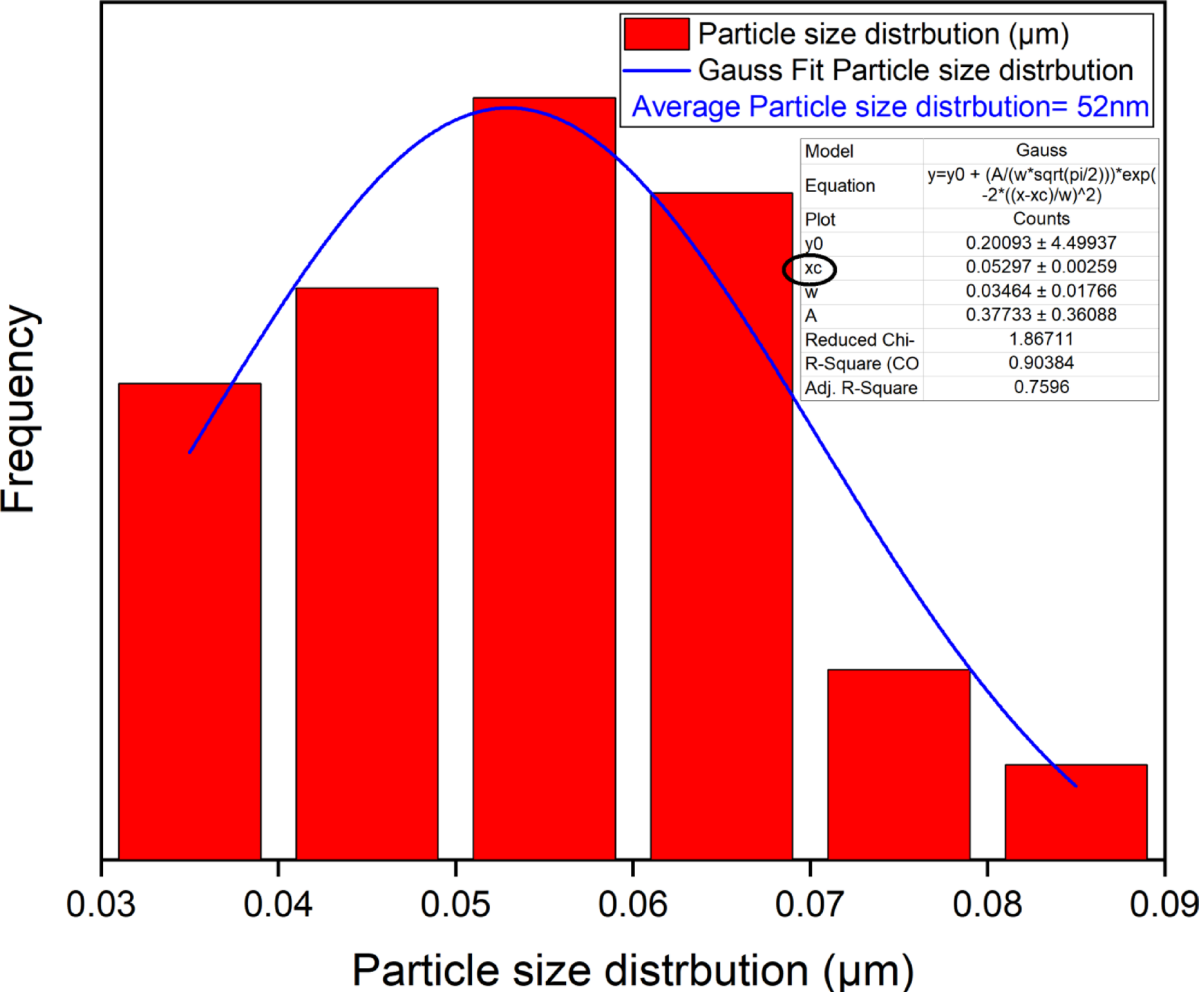
SEM images, particle size distribution of Ni-BDC-MOF in 2 μm scale images and the Ni-MOF \magnetite in 1 μm scale.

The SEM technique explain the surface and morphology of the adsorbent in addition to it revealed a range of particle sizes which give an image about size of particles even small or large which will effect on the pore size that is critical for the adsorption mechanism in general^1–4^. The morphology and particle size distribution of the Ni-BDC-MOF and the Ni-MOF \magnetite were examined using a scanning electron microscope (SEM) in conjunction with ImageJ software (version 1.53e/Java 1.8.0_172)^1,75^. Figures (2 A and C) suggested that the morphology of the Ni-BDC-MOF and the Ni-MOF \magnetite consists of rods-like structures and sheets with rods like structure for, respectively. Additionally, the analysis of the particle size distribution of the sample was performed utilizing ImageJ software (version 1.53e/Java 1.8.0_172) based on SEM images with1 and 2 μm scales (Fig. 2, B and D). The SEM image revealed a range of particle sizes in the Ni-BDC-MOF structure, with a mean size of particles of between 35 and 75 nm, and Ni-MOF \magnetite having a range of particle size with mean average size 52 nm. These results relive the uniform surface with nanoscale dimensions of the prepared samples^74,76,77^.

#### Transmission electron microscope (TEM)

**Fig. 3:**
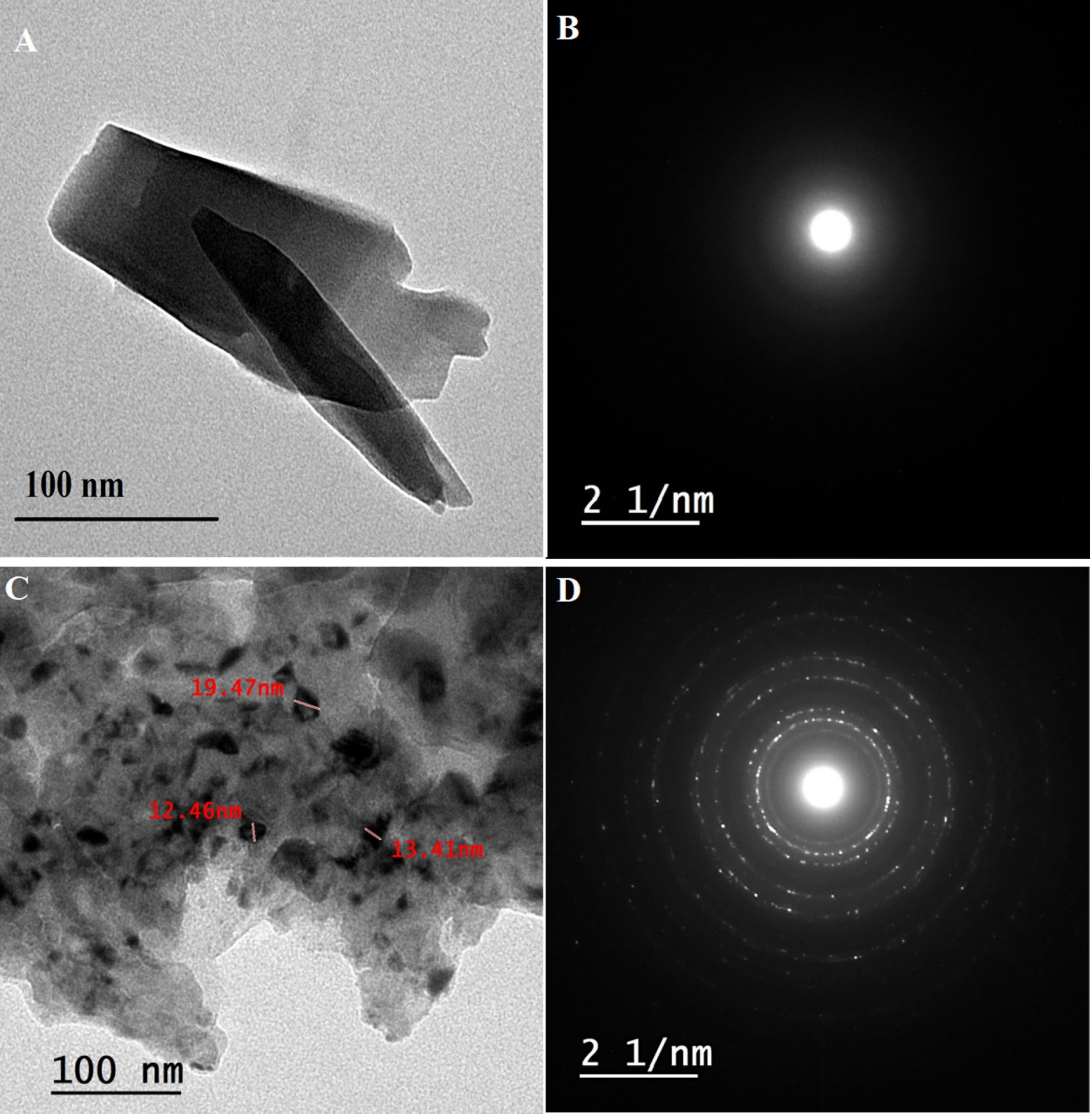
The HR-TEM (**A**) and (**C**) and SAED (**B**) and (**D**) of Ni-BDC-MOF and the Ni-MOF \magnetite, respectively.

The transmission electron microscopy (TEM) image (Fig. 3A, C) showed that the crystal structure of Ni-BDC-MOF is shaped like sheets and rods. Ni-MOF magnetite nanoparticles, on the other hand, displayed particles with uneven shapes. TEM investigation also revealed the particle sizes of Ni-BDC-MOF and Ni-MOF magnetite nanoparticles, which ranged from 36.42 to 72.2 and 12.46 to 19.49 nm, respectively. As demonstrated in SAED, which suggests selected area electron diffraction, the high-resolution transmission electron microscope image (TEM) of Ni-BDC-MOF and Ni-MOF magnetite nanoparticles (Fig. 3B and D) verified the good crystallinity of the produced nanoparticles^2,3,45^.

#### Brunauer-Emmett-Teller (BET)

The nitrogen adsorption (BET) study clarified that the surface area of Ni-BDC-MOF and the Ni-MOF \magnetite nano particles were found to be 432.31 and 373.27 m^2^/g, indicating an elevated surface area of the synthesizes samples. Furthermore, the average pore sizes are 6.27 and 4.75 nm indicating that the Ni-BDC-MOF and the Ni-MOF \magnetite nano particles have mesoporous structures, which was also previously demonstrated by XRD data^74,76,77^.

### The factors affecting the adsorption process

Research has been done on the factors influencing dye removal from aqueous solutions, such as pH, adsorbent dosage, initial dye concentration, stirring rate, volume, and contact duration, to identify the ideal parameters suggesting the maximum possible removal efficacy of Rhodochrome under study.

#### Effect of pH

**Fig. 4:**
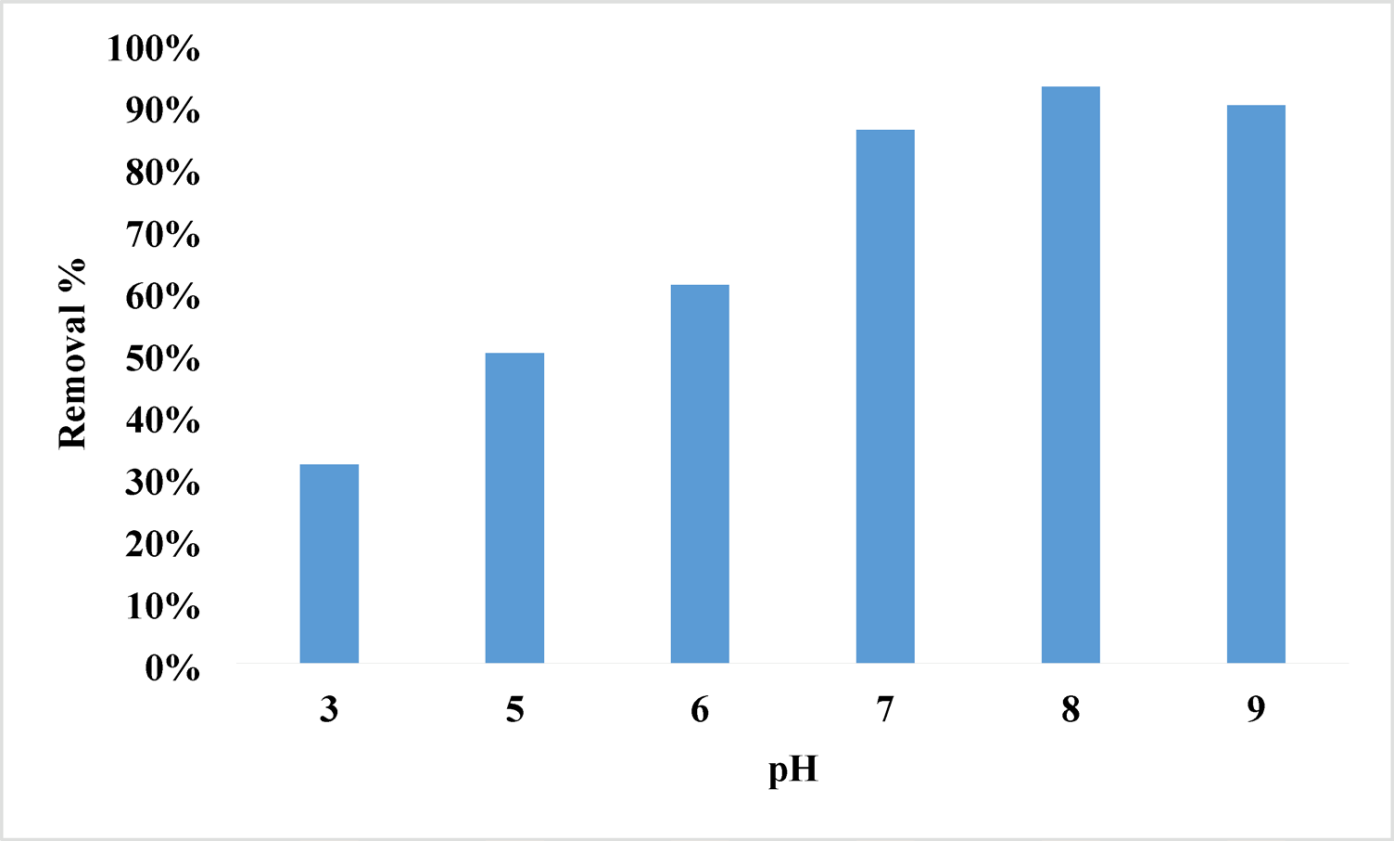
The effect of pH on the removal of Rhodochrome.

**Fig. 5:**
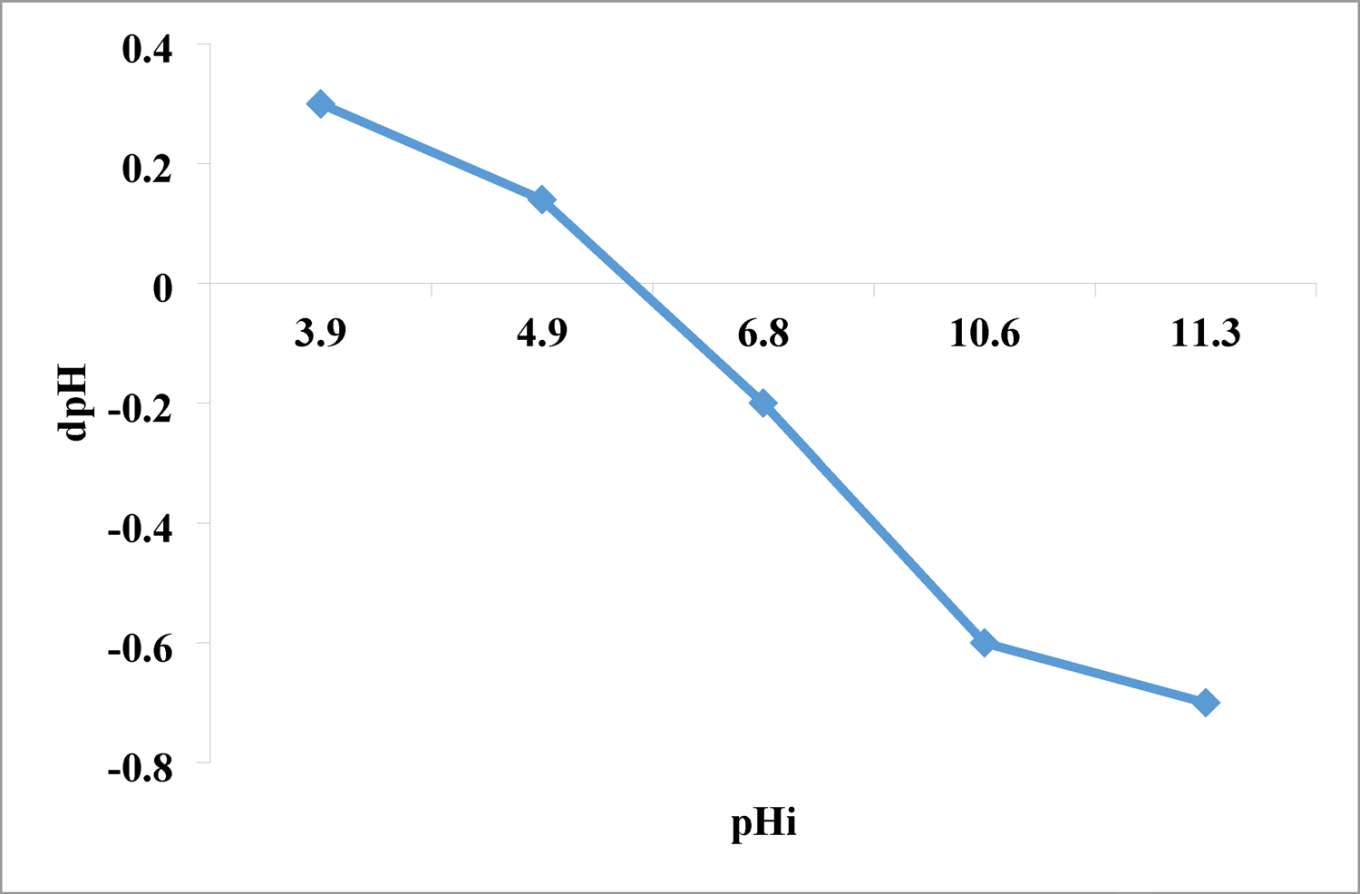
The point of zero charge (pH_pzc_) of the NPs.

The pH of the dye solution must be considered while assessing dye adsorption. The surface charge of the adsorbent or the speciation of the adsorbate may be associated with this effect. The influence of pH on the adsorption capacity of Ni-MOF/Magnetite NPs for rhodochrome was examined across a pH spectrum of 3 to 9. The properties of the material being removed (whether anionic or cationic) and the interaction of the NPs both influence the impact of pH on adsorption. The results presented in Fig. 4 illustrate that the adsorption process is more effective in basic conditions. 5.8 is the pH at which the surface charge of nanoparticles becomes neutral (pH_pzc_) shown in Fig. 5. When the pH_pzc_ is below it, the surface has a positive charge which will able to attract the negative adsorbates; when it is above it, a negative charge which will attract positive adsorbates as rhodochrome. This causes a significant electrostatic pull between the cationic rhodochrome and the nanoparticles, hence promoting their contact^78–81^. 10 ppm of cationic rhodochrome using a dose of 0.07 g after 5 min, the best removal occurred between a pH ranges of 7 to 9, with a maximum removal rate of 93% at a pH of 8.

#### Effect of adsorbent dosage

**Fig. 6:**
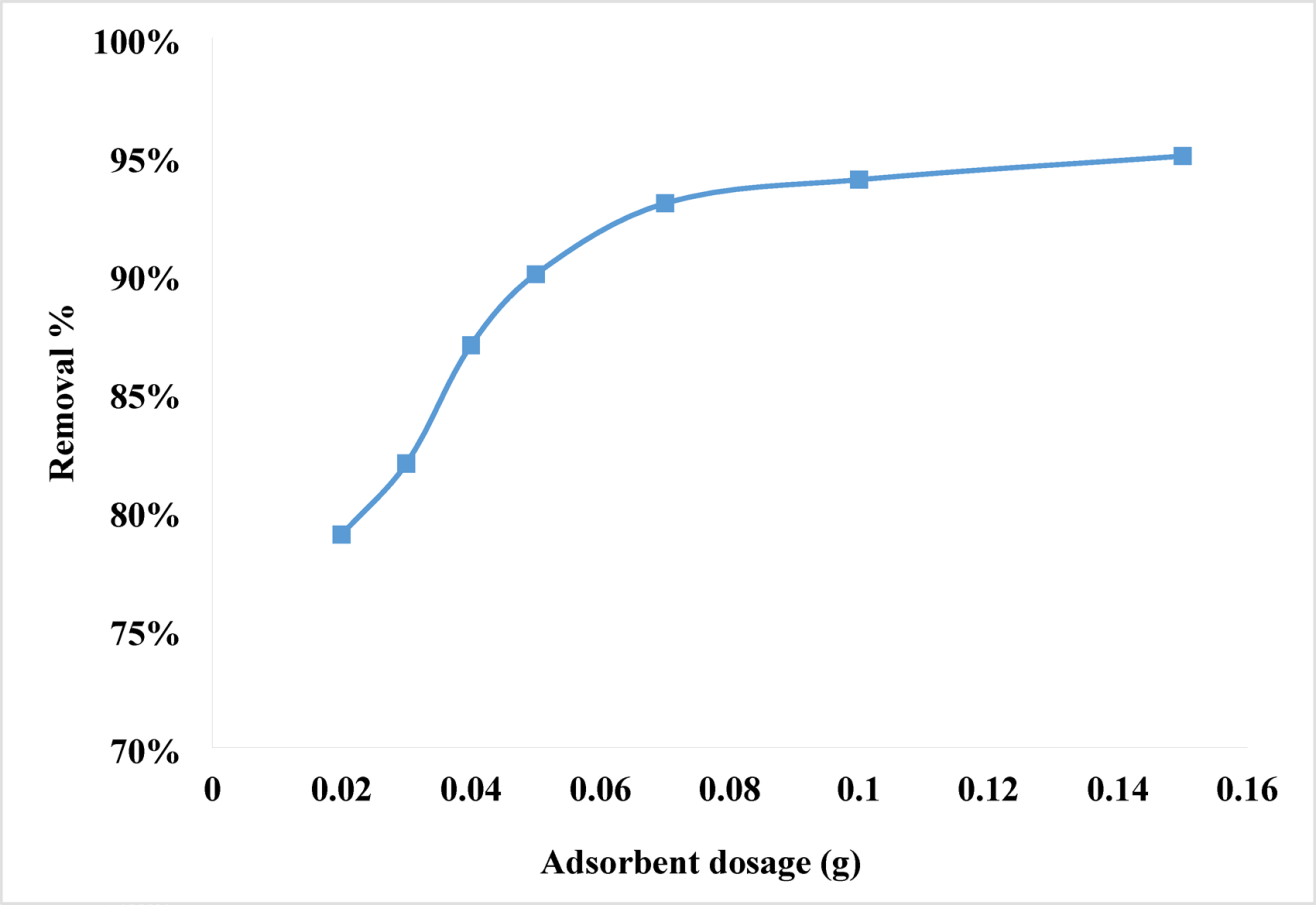
The impact of varying the NPs dosage on the elimination of Rhodochrome.

The effect of adsorbent dose was investigated by varying the quantity of NPs from 0.02 to 0.15 g/100 mL. This was done while keeping the initial concentration at 10 ppm and the agitation of the adsorbent at 600 rpm. This circumstance is illustrated in Fig. 6. When the amount of absorbent in the solution increased from 0.02 to 0.15 g, the removal percentage increased from 79 to 95%. This was the outcome of the increase. When the dosage of the adsorbent is increased, the number of active sorption sites on its surface increases, which results in an increase in the proportion of the substance that is removed^82–85^. Regarding this, it is clear why it results in improvement. After five minutes, the optimal dosage was determined to be 0.07 g, with elimination rate of 93%.

#### Agitation speed effect

**Fig. 7:**
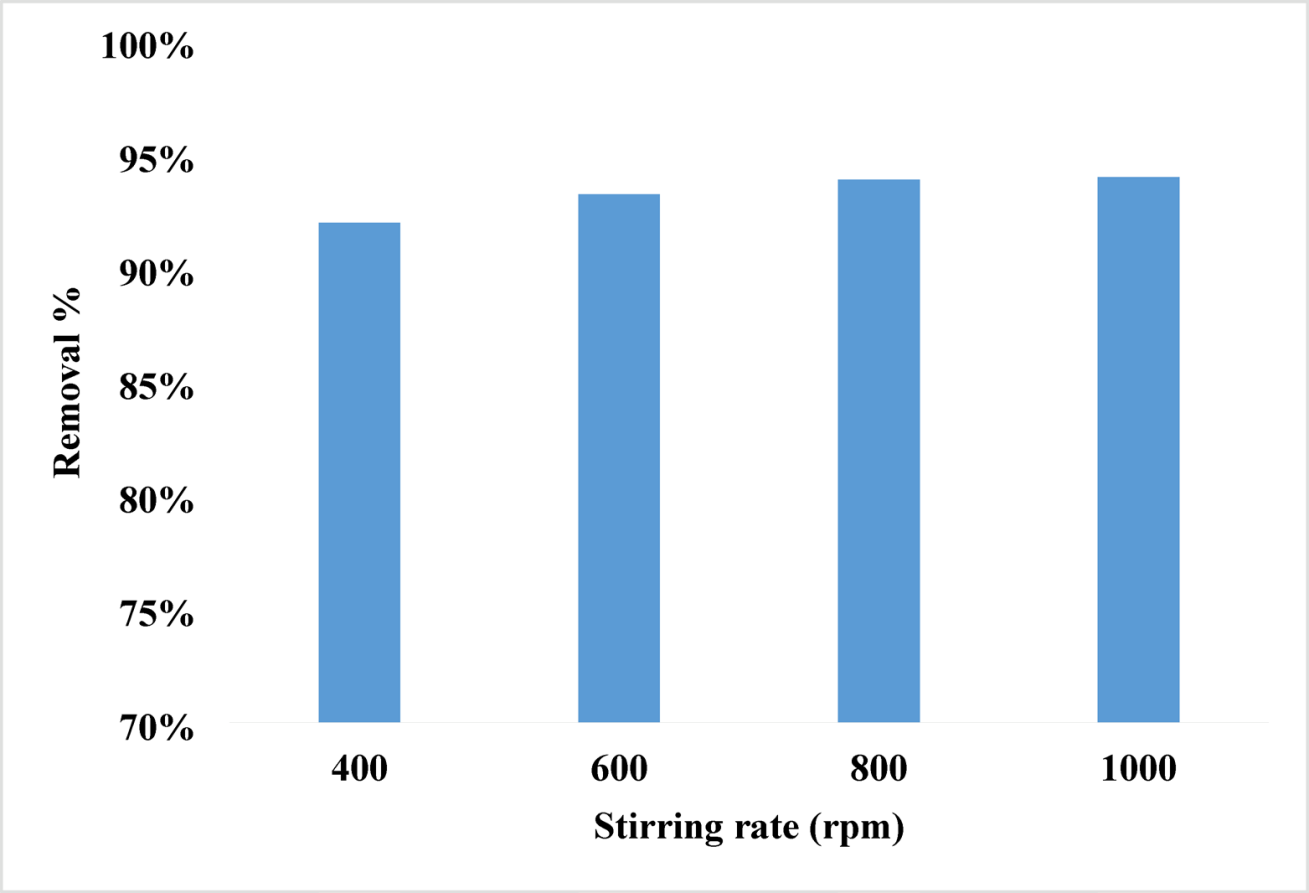
The effect of stirring rate on the removal of the rhodochrome.

Between 400 and 1000 rpm, the removal rate rises from 92% at 400 rpm to its optimal of 94% at 1000 rpm. That is attributed to increase the kinetic energy among molecules and therefore increases collisions and lowers boundary layer resistance, may cause this effect. Moreover, higher agitation speed reduces the effect of external mass transfer, hence encouraging closer interaction between the adsorbent and the adsorbates phase^86–89^. Figure 7 shows 10 ppm rhodochrome with 0.07 g of NPs at pH 8 against various agitation speeds which explains the adsorption rate is not much influenced by agitation speed.

#### Effect of initial dye concentration

**Fig. 8:**
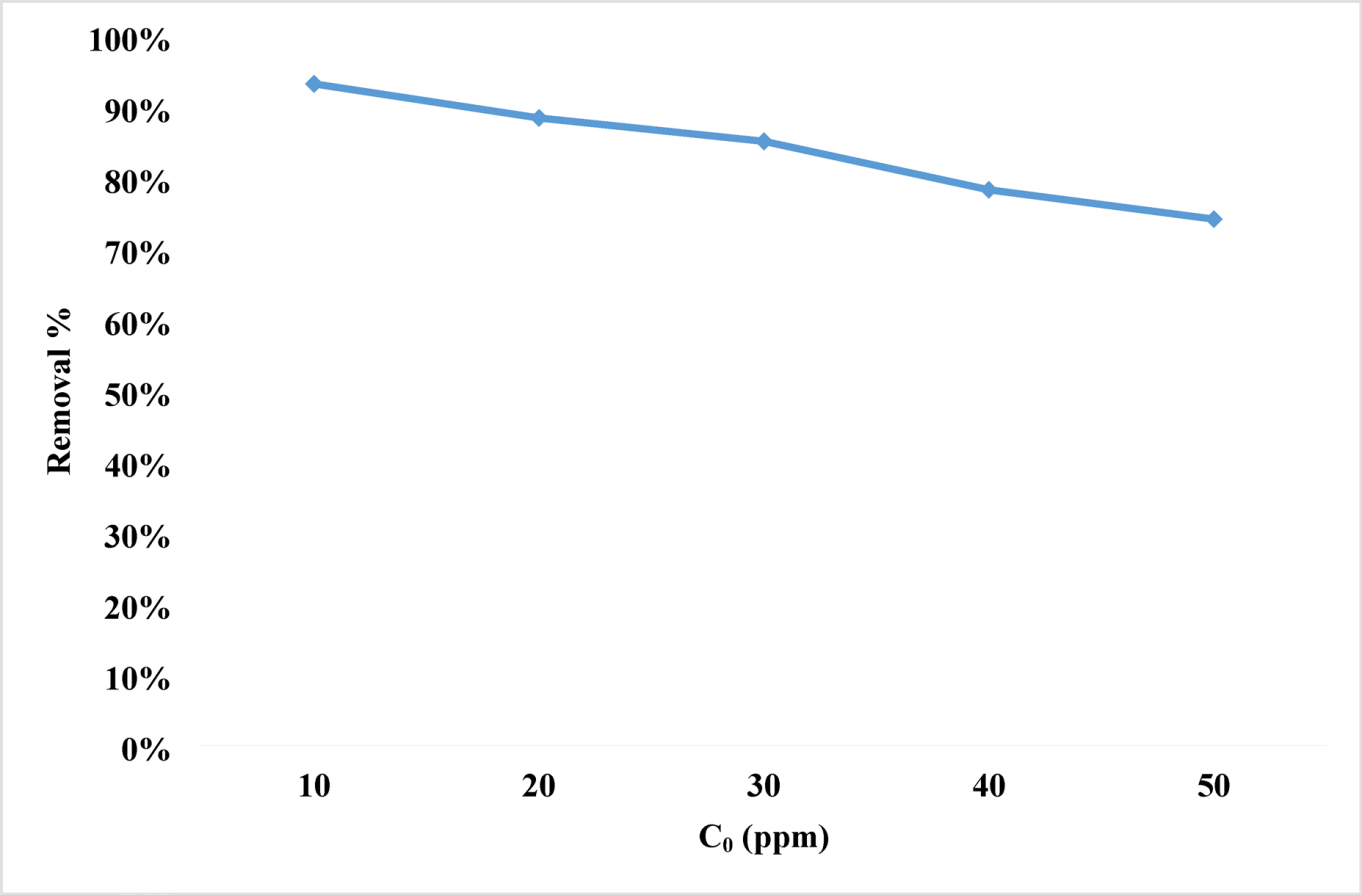
Removal efficiency of different concentrations of rhodochrome dye.

One common definition of dye adsorption, a mass exchange process, is the accumulation of dye at the interface of the liquid-solid phase. The initial concentration of the dye gives the necessary push to overcome the mass transfer barrier from the liquid to the solid phase. The driving force improves with increasing initial concentration. Regarding the influence of the initial rhodochrome concentration, it is evident from Fig. 8 that the removal percentage decreases with increasing initial concentration. With an increase in initial dye concentration from 10 to 30 ppm, the removal percentage falls from 93% to 85%, and with an increase from 30 to 50 ppm, it falls from 85% to 74%. The percentage of dye removed dropped due to a reduction in the number of active sites on the adsorbent, which was caused by an increase in the initial dye concentration. This is notably obvious when rhodochrome is present at more than 50 ppm. The mass transfer barrier between the liquid phases (dye, water, and other interferences) and the solid phase (NPs. adsorbent) also lowers removal %. As the initial concentration decreased, the barrier to mass transfer decreased, improving dye removal efficiency^90,91^.

#### Effect of contact time

**Fig. 9:**
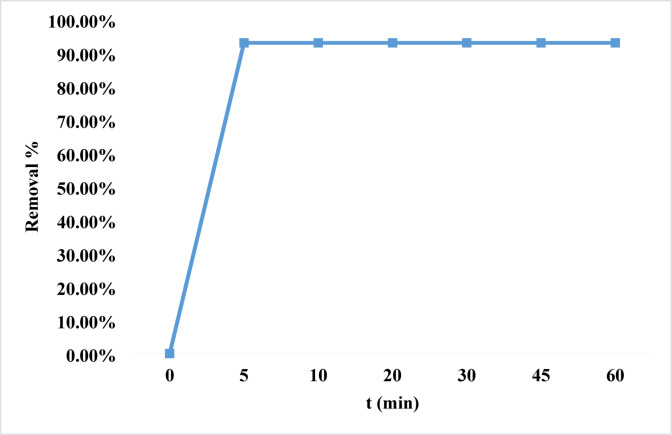
The effect of contact time on the removal of the rhodochrome dye.

An effective wastewater treatment system depends on the time at which equilibrium is attained. Adsorption kinetics is a crucial measure to examine in the framework of knowing the adsorption process and evaluating the effectiveness of the adsorbent. A rapid and precisely monitored adsorption rate is required for a substance to be successful as an adsorbent. With time intervals from 5 min to 60 min, a study was done to investigate how contact duration affects dye adsorption. After just five minutes, the elimination percentage at pH 8 and dye concentration of 10 ppm is about 93% and it remains unchanged at any other time. This is demonstrated in Fig. 9^78–91^.

### Adsorption isotherm

Adsorption isotherm models can be created by assuming a thermodynamic equilibrium between the quantity of adsorbed molecules per unit mass of adsorbent and the amount of adsorbate in the bulk fluid phase at a fixed temperature and pH. It gives information about the distribution of adsorbable solutes in liquid and solid phases at different equilibrium concentrations. also generate different parameters for each system. The Langmuir, Freundlich, Temkin, and Dubinin-Radushkevich (D-R) isotherms are mathematical models used to describe how adsorbates (molecules or atoms) interact with adsorbents (solid surfaces) during adsorption. Each model is based on various assumptions about the adsorption and surface processes. They were also used to test adsorption isotherms for the treatment of water and wastewater^92^.

#### Langmuir isotherm

The Langmuir isotherm remains the most fundamental and effective method for both physical and chemical adsorption. This model assumes monolayer adsorption, where just a single layer of molecules is absorbed, the adsorbent surface is homogeneous, adsorption energy is uniform across all sites, and there is no adsorbates transmigration along the surface plane. Adsorption on the surface is localized, meaning atoms or molecules are adsorbed at specific locations once a contaminant is present^92^.

Equation (3) represented the Langmuir isotherm based on these assumptions (Eq. 3).

3 $$\:q_{e} = q_{{\max }} *\frac{{bc_{{e\:}} }}{{1 + bc_{{e\:}} }}\:\:$$

The linear form is the following (Eq. 4):

4 $$\:\frac{{c_{e} }}{{q_{e} }} = \frac{1}{{q_{{\max }} *b}} + \left( {\frac{1}{{q_{{\max }} }}} \right)c_{{e\:}} \:\:$$

**Fig. 10:**
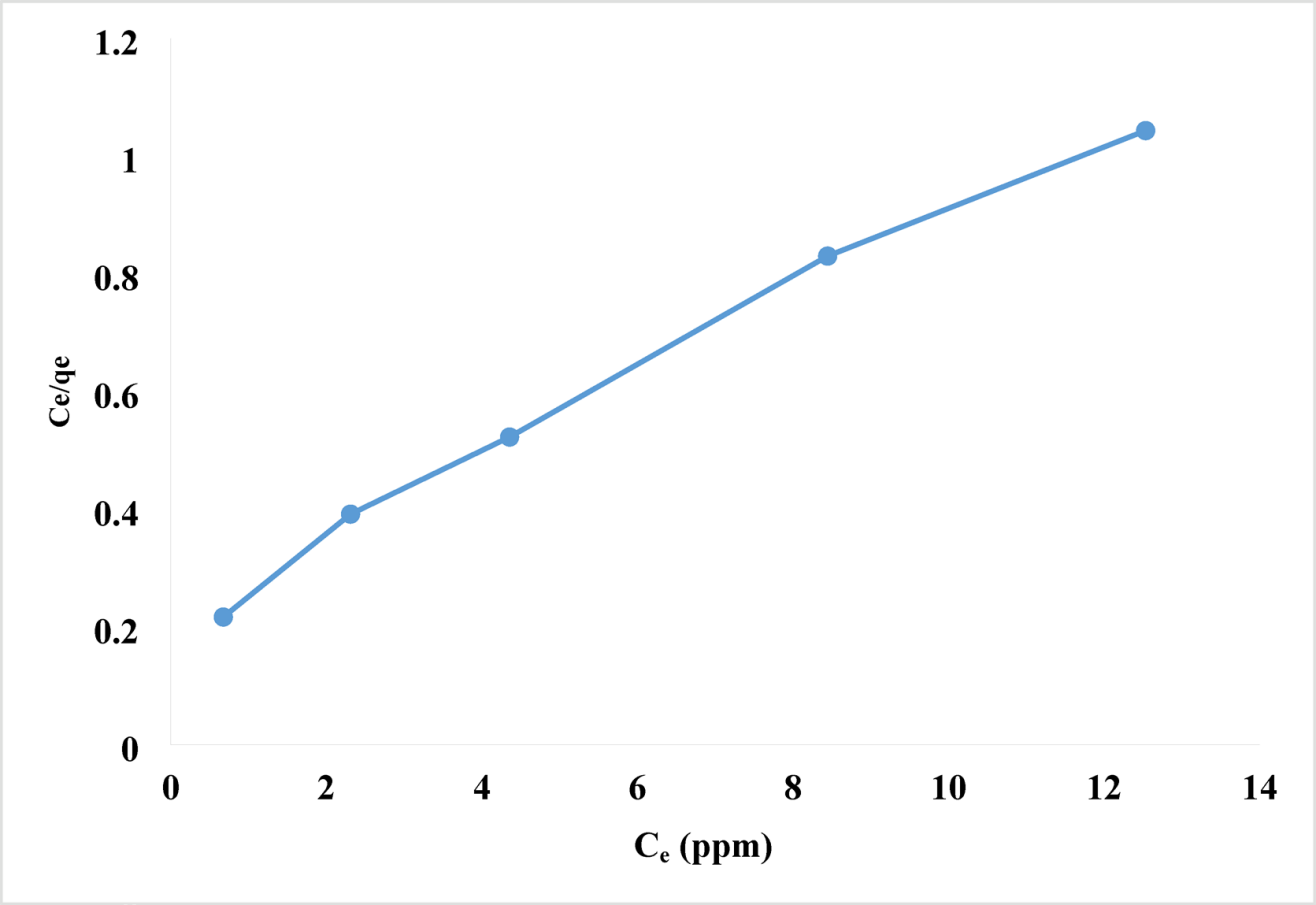
Langmuir adsorption isotherm of rhodochrome onto Ni-MOF/Magnetite NPs.

Where, q_e_ represents the milligrams of adsorbate per gram of sorbent, q_max_ (in mg/g) denotes the adsorption capacity, C_e_ (in mg/L) indicates the concentration of the equilibrium solution phase, and b (in L/mg) is the affinity coefficient, which correlates with the energy of adsorption^93^. The values of q_max_ and b were derived from the analysis of the linear correlation between C_e_/q_e_ and Ce, as illustrated in Table 2; Fig. 10.

The maximal adsorption capacity of rhodochrome per gram of nanoparticle was discovered to be 14.50 mg, and the Langmuir constant equilibrium b, was calculated to be 0.33. The R^2^ value of 0.98 demonstrated a close fit between the sorption data and the Langmuir Isotherm model. The separation factor (R_L_) for the highest amount of adsorbate (C_o_) in mg/L was calculated using Eq. (5) to verify the Langmuir isotherm’s fundamental features.

5 $$\:R_{L} = \frac{1}{{1 + bC_{ \circ } }}$$

R_L_ discusses the shape and favorability of adsorption between adsorbents and Rhodochrome. The value of R_L_ determines whether the isotherm is favorable (0 < R_L_ < 1), unfavorable (R_L_ > 1), linear (R_L_ = 1), or irreversible (R_L_ = 0)^94^. Table 3 showed the R_L_ constant value for rhodochrome adsorption by NPs, which indicates that the approach is favorable.

**Table 2:** Langmuir, Freundlich, TEMKIN models results and R^2^ values of rhodochrome adsorption on NPs.

Isotherm	Results
Langmuir isotherm	q_max_ = 14.50 mg/g b = 0.33 L/mg R^2^ = 0.9897
Freundlich isotherm	K_f_ = 4.323 (mg/g) (L/g)^*1/n*^ *n* = 2.45 1/*n* = 0.408 R^2^ = 0.984
Temkin isotherm	B = 3.49 J/mol b = 708.69 J/mol A = 2.364 L/mg R^2^ = 0.9928
Dubinin-Radushkevich isotherm	E = 0.767 KJ/mol q_max_ = 11.285 R^2^ = 0.9389

**Table 3:** Values of separation factor (R_L_).

C_o_ (mg/L)	*R*_L_
10	0.23
20	0.13
30	0.09
40	0.07
50	0.06

#### Freundlich isotherm

A Langmuir-like empirical equation that can be used to model multilayer adsorption is the Freundlich isotherm model. According to this hypothesis, active sites and energy are spread exponentially across the heterogeneous surface of the adsorbent. Adsorption of energy decreases exponentially at the end of the adsorption phase, with the strongest binding sites being occupied first. The expression for the Freundlich isotherm is Eq. (6).

6 $$q_{e} = K_{f} C_{e}^{{(1/n)}}$$

To linearize Eq. (6), use the following constants and logarithms:

7 $$\:{log}{q}_{e}=log{k}_{f}+\frac{1}{n}{log}{C}_{e}$$

**Fig. 11:**
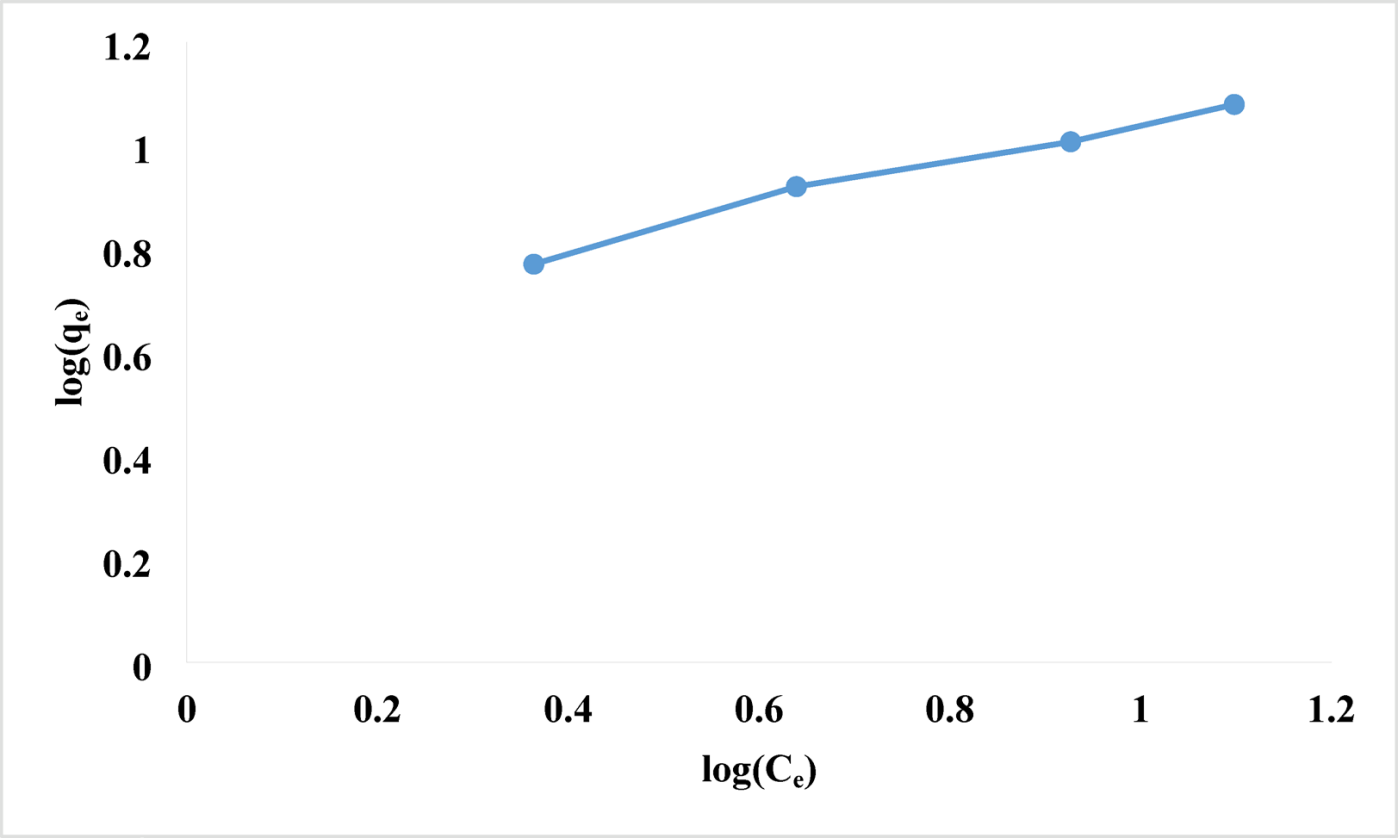
Freundlich adsorption isotherm of rhodochrome dye onto NPs.

Adsorption coefficient k_f_ is the adsorbent’s capacity, measures the adsorbate’s ability to adhere to the adsorbent. 1/n indicates adsorbate adsorption intensity or surface heterogeneity. A slope (1/n) between 0 and 1 indicates a favorable adsorption isotherm. The 1/n above one suggested unfavorable adsorptive isotherms, while closer to zero indicates a heterogeneous adsorbent surface and nonlinear isotherm. Higher K_f_ values result in increased adsorption intensity^94,95^. As shown in Table 2; Fig. 11, the Freundlich isotherm showed that rhodochrome dye adsorption onto NPs is favorable and strongly bound to the surface, while values of n between 1 and 10 showed that rhodochrome favors NP adsorption.

#### Temkin isotherm

According to the Temkin isotherm equation, the heat of adsorption for each molecule in the layer decreases linearly as coverage rises due to interactions between the adsorbent and the adsorbate. Furthermore, assumes that the adsorption process has a uniform distribution of binding energies up to a given maximal binding energy (Eq. 8).

8 $$\:{q}_{e}=\frac{RT}{b}{ln}A+\frac{RT}{b}{ln}{C}_{e}$$

The equilibrium binding constant, A (L/mol), indicates the maximal binding energy. B is the adsorption heat constant, defined as B = RT/b, where R is the universal gas constant (8.314 J/mol·K), T is the temperature (298 K), and b is a constant related to the heat of sorption (J/mol) derived from the Temkin plot of qe vs. ln Ce^95,96^.

**Fig. 12:**
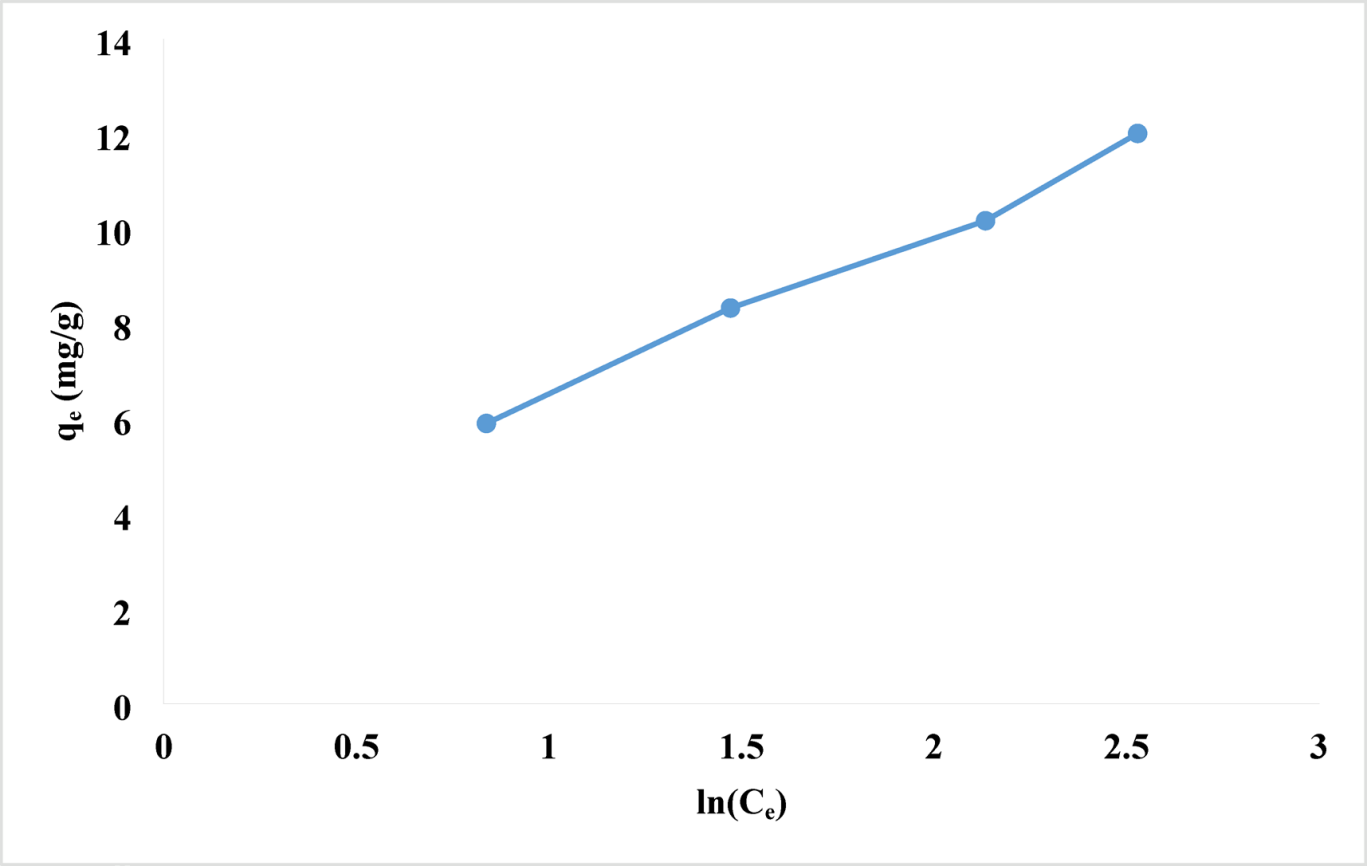
Temkin adsorption isotherm of rhodochrome dye onto NPs.

As seen in Fig. 12, the Temkin isotherm was formed as a straight line. The Temkin isotherm constant (b) can be calculated using the slope, B = RT/b, and the intercept, (RT/b) lnA, as shown in Table 2.

#### Dubinin-Raduskevich (DR) isotherm

The Dubinin-Radushkevich isotherm model is an empirical model originally formulated for the adsorption process via a pore-filling mechanism. This term is frequently employed to characterize the adsorption process that takes place on both homogeneous and heterogeneous surfaces. The DR isotherm model was preferable than the Langmuir isotherm since it did not consider a uniform surface or a constant adsorption potential. Equations (9–11) presented the nonlinear representation of the DR isotherm model^97^.

9 $$\:{ln}{q}_{e}={ln}{q}_{max}-\beta\:{\epsilon\:}^{2}$$

10 $$\:\epsilon\:=RT{ln}\left(1+\frac{1}{{c}_{e}}\right)$$

11 $$\:E=\frac{1}{\sqrt{2\beta\:}}$$

**Fig. 13:**
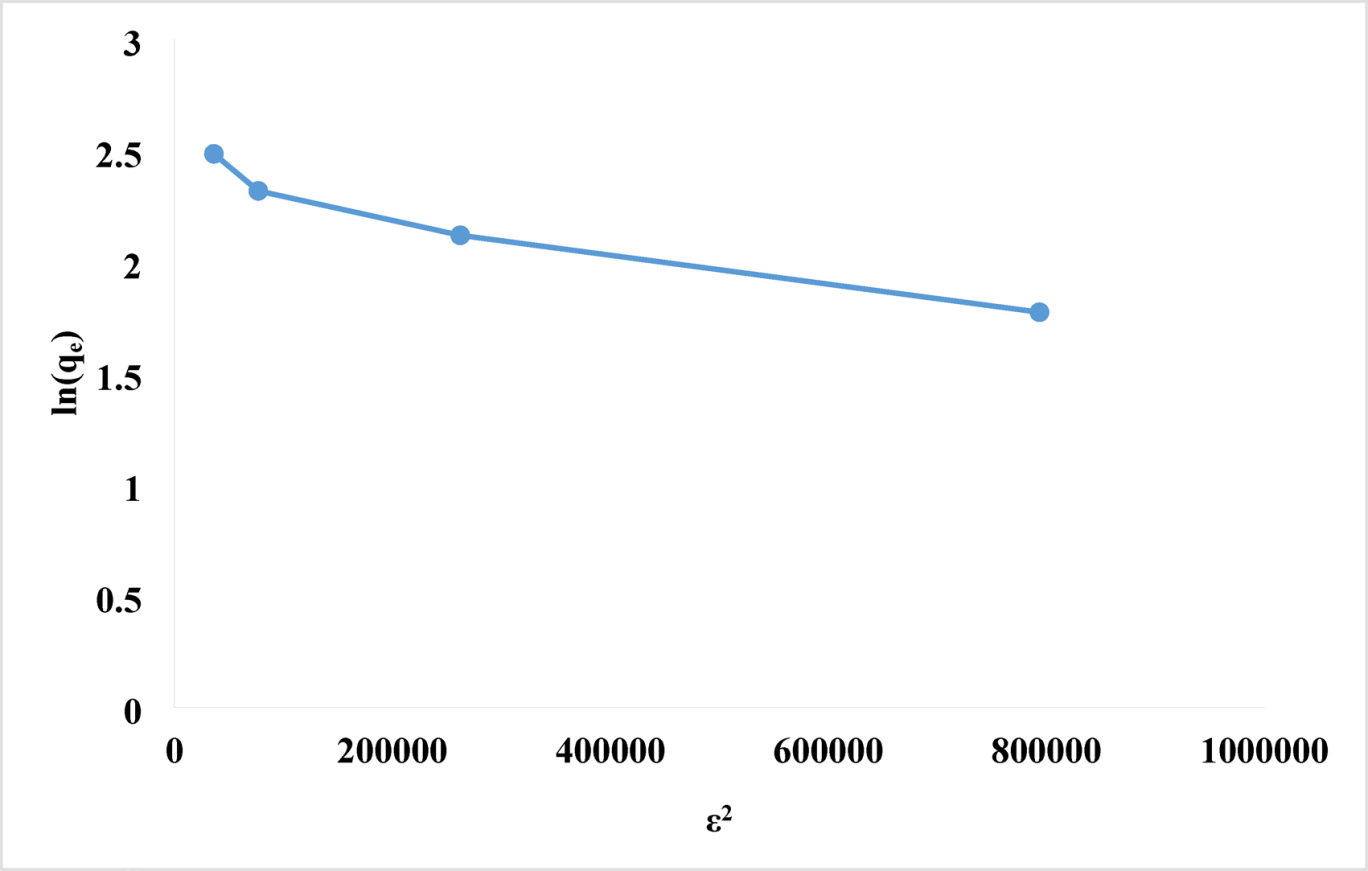
Dubinin-Radushkevich isotherms of rhodochrome dye onto NPs.

The adsorption energy constant is β (mol^2^ J^− 2^), the adsorption potential is ε (J mol^− 1^), the universal gas constant is R (J mol^− 1^ K^− 1^), the absolute temperature is T (K), and the unit of Ce and Cs is mol L^− 1^. The nature of the adsorption process is mostly determined by the mean free energy (E): if 8 < E < 16 kJ/mol, the process is ion exchange; if E < 8 kJ/mol, it is physical interaction; and if E > 16 kJ/mol, it is chemical interaction^98^. E = 0.767 kJ/mol, indicated physical contact in the adsorption process. Figure 13; Table 2 provided straight lines from the D-R isotherm plotting ln q_e_ against ɛ^2^ with E = 0.767 kJ/mol, q_max_ = 11.285 mg/g, and R_2_ = 0.9389. Based on the Dubinin-Radushkevich isotherm, the mean adsorption energy indicates physisorption, not chemisorption. Physisorption controls the adsorption process, as shown by an E value below 8 kJ/mol.

According to the study of the isotherm’s experimental data (Table 2). Langmuir, Freundlich and the Dubinin-Radushkevich isotherm give the least precise match. Nonetheless, the Temkin model has the highest correlation coefficient (0.9928) of the models used. This indicates that the model may accurately represent rhodochrome dye adsorption onto Ni-MOF/Magnetite NPs.

### Adsorption kinetics

**Fig. 14:**
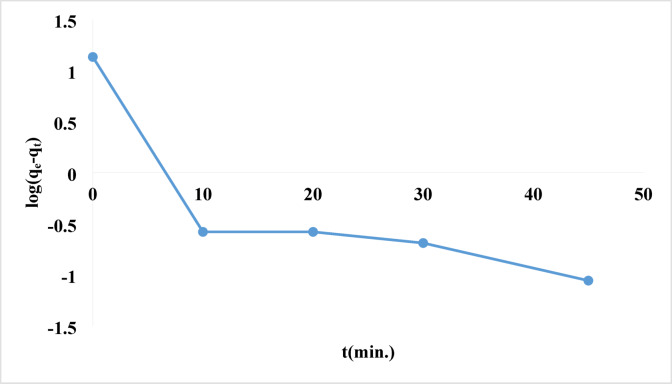
Pseudo-first order kinetics of the removal of rhodochrome dye.

**Fig. 15:**
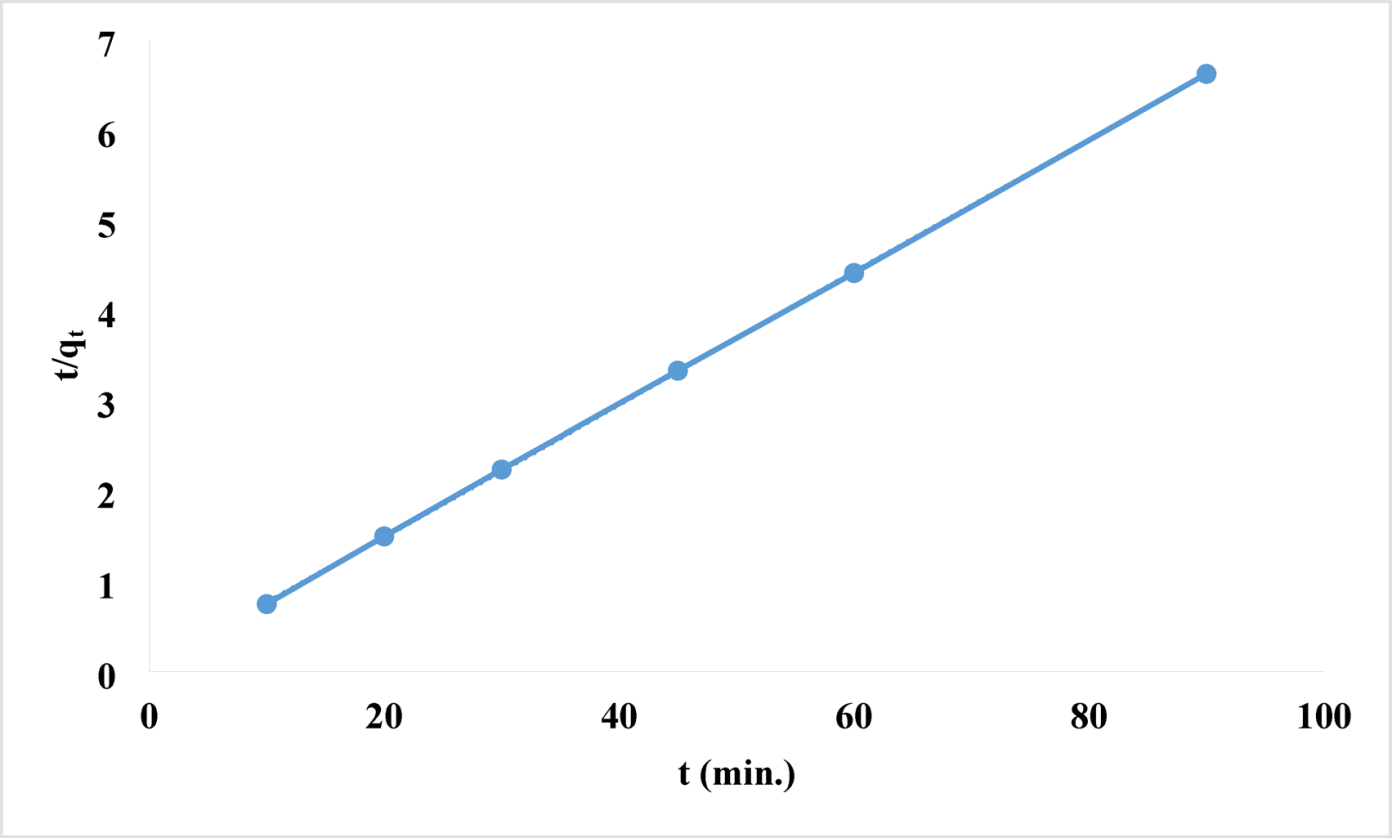
Pseudo-second order kinetics of the removal of rhodochrome dye.

Adsorption kinetic models provide useful information on how rapidly pollutants cling to surfaces. These models are used to illustrate experimental data to determine how pollutants are adsorbed from aquatic systems at the adsorbent-adsorbate interface. We investigated the kinetic data using pseudo-first order and pseudo-second order models to determine the adsorption mechanism. Equations (12) and (13) were used for this:

12 $$\:{log}\left({q}_{e}-{q}_{t}\right)={log}{q}_{e}-\left(\frac{{k}_{1}}{2\cdot\:303}\right)t$$

13 $$\:\frac{t}{{q}_{t}}=\left(\frac{1}{{k}_{2}{q}_{e}^{2}}\right)+\left(\frac{1}{{q}_{e}}\right)t$$

where k_1_ and k_2_ are the first- and second-order rate constants, respectively, and qe and qt are the amounts of rhodochrome dye adsorbed on the surface of the Ni-MOF/Magnetite NPs at equilibrium and time (t), in mg/g^99,100^. The pseudo-first-order module indicates the amount of adsorbate that has accumulated on the adsorbent’s surface over time. It is consistent with the reaction if one or more concentrations have a significant effect on the rate at which the reaction occurs. It largely describes the adsorption that occurs on surfaces with heterogeneous adsorbents. While the pseudo-second-order module was designed with the goal of imitating the actual reaction as accurately as possible. A chemisorption process is said to be the slowest of all processes^101^. Table 4 enumerated and defined each of the parameters. Figure 14 illustrated low correlation values in the pseudo-first-order kinetic model (R² = 0.6577). Moreover, a notable disagreement existed between the empirical and theoretical results regarding the equilibrium adsorption capacity (q_e_), indicating that the pseudo-first-order model inadequately represented the experimental data. In contrast, the results of pseudo-second-order kinetics demonstrated that a linear fit was attained with exceptionally high correlation coefficients (R^2^ = 1), as illustrated in. Figure 15. In pseudo-second-order kinetics, theoretical q_e_ values align closely with the experimental results.

**Table 4:** The kinetic parameters for rhodochrome dye adsorption on NPs.

Kinetic model	Parameters	Results
pseudo- first order	q_e_(exp.) mg/g	13.58
	q_e_(theo.) mg/g	3.009
	K_1_ (min^− 1^)	0.09
	R^2^	0.6577
pseudo-second order	q_e_(exp.) mg/g	13.58
	q_e_(theo.) mg/g	13.65
	K_2_ (min^− 1^)	0.17
	R^2^	1
intra-particle diffusion	C (mg/g)	5.00
	Ki (mg/g.min^1/2^)	1.23
	R^2^	0.576

#### Intra-particle diffusion

**Fig. 16:**
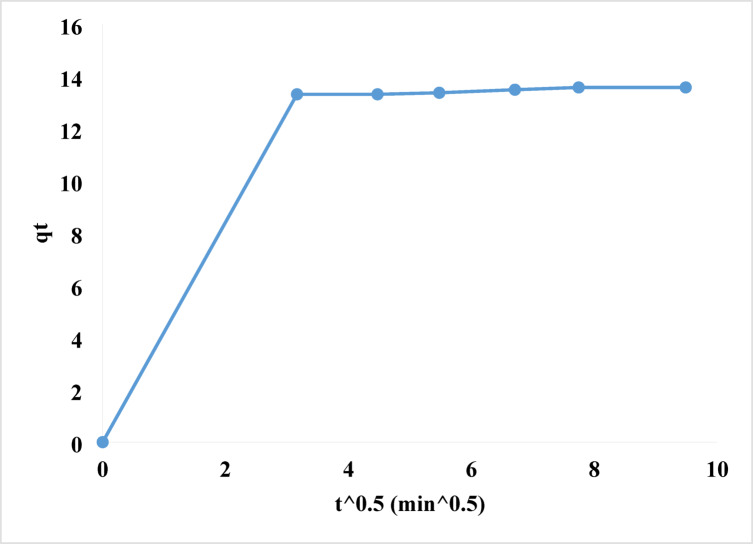
Intra-particle diffusion kinetics of the removal of rhodochrome dye.

An intra-particle diffusion model is frequently employed to assess the rate-regulating process involved in adsorption. The rhodochrome movement within adsorbent particles’ pores determines the adsorption rate, according to this concept. This model is essential for understanding liquid adsorption’s rate-determining step. In batch processes, rate-determining steps may involve solute diffusion from bulk solution to solid surface (intra-particle diffusion) or diffusion over the solid surface’s boundary layer (film)^102^. Intra-particle diffusion can be estimated using Eq. (14):

14 $$\:{q}_{t}={k}_{i}{t}^{0.5}+C$$

q_t_ represented rhodochrome dye adsorption (mg/g) at time t, k_i_ represents intra-particle diffusion rate (mg/g.min^1/2^), and C represents boundary layer thickness (mg/g)^103^. If intra-particle diffusion affects rate, the plot of q_t_ vs. t^0.5^ should be linear, crossing the origin with a zero interception. A divergence from linearity indicates that the rate-determining step should be regulated by boundary layer diffusion^104^. In Fig. 16; Table 4, the connection between q_t_ and t^0.5^ should be straight, as predicted by k_i_ = 0.04 mg/g.min^1/2^, C = 4.40 mg/g, and R^2^ = 0.7245. It does not intersect the zero point. Bounded layer (film) diffusion controls rate determining step.

Based on Figs. 14, 15 and 16; Table 4, it was determined that adsorption kinetics would follow pseudo-second-order kinetics with a high regression R^2^ value of unity. The projected qe values from this rate model show a strong agreement with experimental qe values.

### Validation of the regression coefficient (R^2^)

In a regression model, the coefficient of regression (R²) express how much of the variance dependent variable is explained by the model. But R² alone can be misleading if it is not validated properly. Here are ways to validate regression coefficients R² as adjusted R², cross-validation, test-set performance, coefficient significance, and residual diagnostics.

i. Check Adjusted R² which depends on the value of Adjusted R², which penalizes unnecessary variables. A big difference between R² and Adjusted R² suggests overfitting and vice versa.
ii. Check Statistical Significance of coefficients in which they run t-tests. Low p-values (< 0.05 usually) indicate the coefficient is statistically significant. This helps validate whether predictors are genuinely useful.
iii. Residual Analysis by Plotting residuals vs. predicted values. They should look like random noise (no clear pattern). Patterns suggest model misspecification → invalid R².

All of these parameters can be done automatically by using EXCEL program and ANOVA or by calculation manually. Validate regression coefficients using ANOVA (Analysis of Variance) put all these steps into one clean function and get a validation report about R², adjusted R², p-values and residual plot^105–107^. By applying the factors that have a critical effect on the efficiency of the adsorption removal % as pH, time (min) and amount of dosage (g) to study the statistical factors for the data as R², adjusted R², p-values and residual plot and so on, where pH 8, a duration of 5 min, and 0.07 g of nanoparticles, resulting in a clearance rate of 93%.

**Table 5:** Analysis of variance by ANOVA for response surface.

Source	Sum of Squares	df	Mean Square	F Value	*p*-value Prob > F	
Model	3646.08	9	405.12	412.49	< 0.0001	Significant
*A-pH*	*3362.00*	*1*	*3362.00*	*3423.13*	*< 0.0001*	
*B-Time*	*21.13*	*1*	*21.13*	*21.51*	*0.0004*	
*C-Dosage*	*171.13*	*1*	*171.13*	*174.24*	*< 0.0001*	
*AB*	*0.25*	*1*	*0.25*	*0.25*	*0.6217*	
*AC*	*2.25*	*1*	*2.25*	*2.29*	*0.1524*	
*BC*	*1.00*	*1*	*1.00*	*1.02*	*0.3301*	
*A* ^*2*^	*86.70*	*1*	*86.70*	*88.28*	*< 0.0001*	
*B* ^*2*^	*1.20*	*1*	*1.20*	*1.22*	*0.2876*	
*C* ^*2*^	*1.20*	*1*	*1.20*	*1.22*	*0.2876*	
Residual	13.75	14	0.98			
Std. Dev.	0.99		R-Squared		0.9962	
Mean	68.42		Adj R-Squared		0.9938	
C.V. %	1.45		Pred R-Squared		0.9520	
PRESS	175.57		Adeq Precision		78.551	

As shown in Table 5. Values of “Prob > F” less than 0.0500 indicate model terms are significant. The “Pred R-Squared” of 0.9520 is in reasonable agreement with the “Adj R-Squared” of 0.9938; i.e. the difference is less than 0.2. “Adeq Precision” measures the signal to noise ratio. A ratio greater than 4 is desirable. The ratio of 78.551 indicates an adequate signal. This model can be used to navigate the design space.

**Fig. 17:**
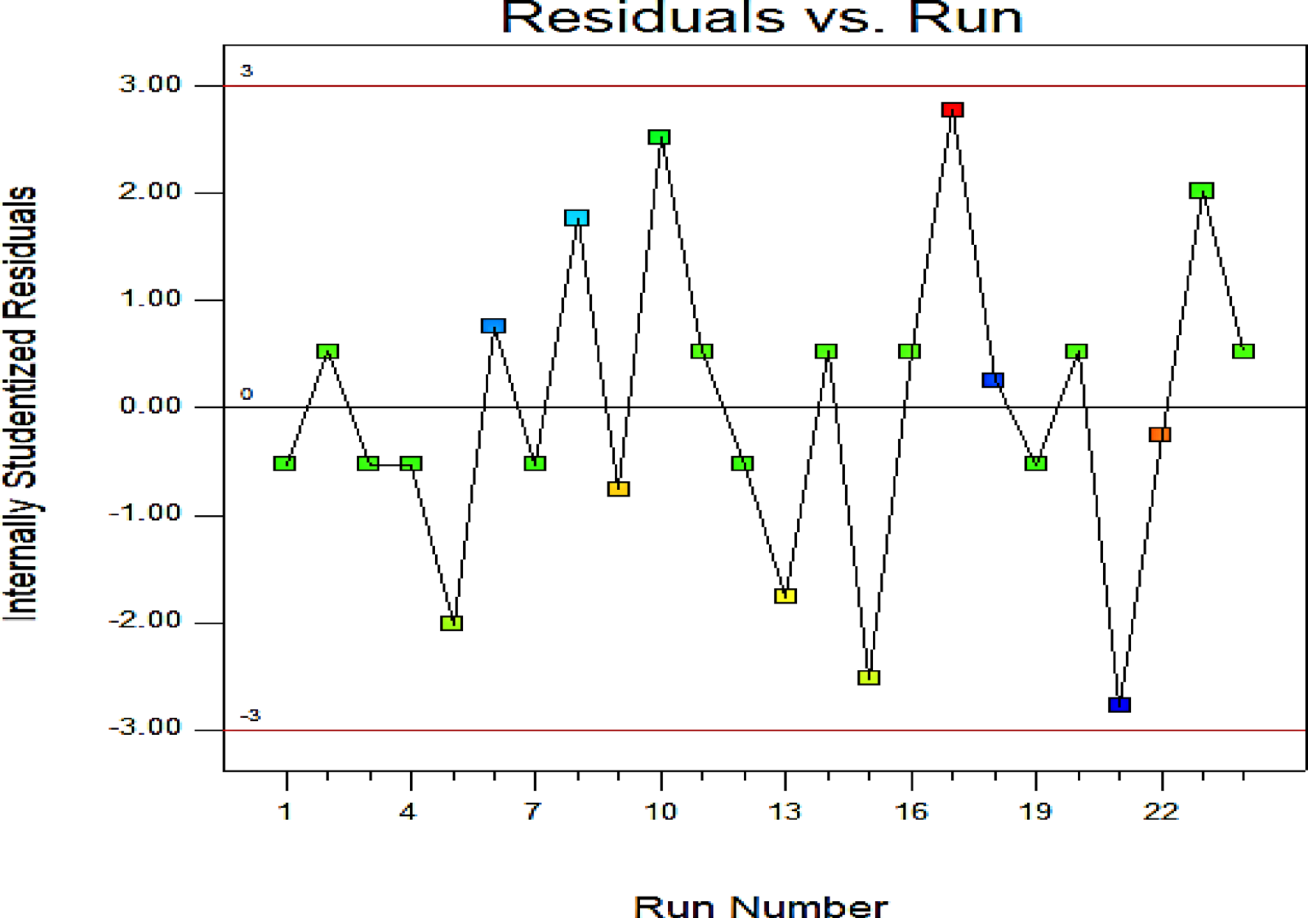
The residual plots.

Also the residuals plots as Fig. 17 give a great indication about the validation of the model specially, they has a relation with the regression coefficient R^2^.

Also the validation of R^2^ can done using excel by data analysis or by calculate the residuals and compare between the computing R^2^ manually using equation R^2^ = 1 - (SSres/SStot) where Total sum of squares (SStot).

Residual sum of squares (SSres) with the R^2^ of the resulting plot.

**Table 6:** Regression statistics.

Parameters	Langmiuer	Freundlich	Temkin	DKR	Intra particle diffusion	Pseudo ^1^st order	Pseudo ^2^nd order
Multiple R	0.990	0.992	0.996	0.968	0.945	0.922	0.999
R Square	0.981	0.984	0.992	0.938	0.894	0.851	0.999
Adjusted R Square	0.972	0.976	0.989	0.907	0.867	0.777	0.999
Standard Error	0.049	0.020	0.271	0.0930	0.044	0.106	0.011
*Significance F*	0.009	0.007	0.003	0.031	0.004	0.077	1.828 × 10^− 10^
R Square manually	0.979	0.971	0.994	0.810	0.403	0.657	0.999
R Square of plot	0.989	0.984	0.992	0.938	0.576	0.657	1.000

As shown in Table 6, If Significance F value < 0.05, the regression model is statistically significant, and your R² is meaningful. In addition to closeness the computing by equation R^2^ with the R^2^ by data analysis EXCEL and R^2^ from the plots which support the validation of the values with low values of standard error. Low significance F values and the closeness the Adjusted R Square with R^2^ calculated support fitting the model with the Temkin and Pseudo 2nd order.

### The adsorption mechanism

The adsorption of cationic Rhodochrome dye onto the Ni-MOF/Magnetite nanoparticle composite is a synergistic process that involves several interactions rather than being controlled by a single mechanism. Electrostatic attraction is the main mechanism, which is firmly supported by pH investigations. Hydrogen bonding, and pore filling/physical adsorption all play important roles.

#### Electrostatic attraction is the main mechanism

The main mechanism for rhodochrome adsorption onto Ni-MOF/Magnetite nanoparticles is electrostatic attraction, significantly affected by the pHpzc of Ni-MOF/Magnetite nanoparticles across different pH levels. This indicates that the adsorbent surface obtains a negative charge at pH levels exceeding 5.8. The rhodochromethes possess a positive charge (R^3+^ cations), enhancing the adsorption process through effective electrostatic attraction between oppositely charged entities. The electrostatic attraction between positively charged Rhodochrome cations (R^3^⁺) and negatively charged nanoparticles significantly enhances removal efficiency, increasing from 32% at pH 3, where repulsion is present, to 93% at pH 8, where maximal attraction occurs.

#### Mechanisms of secondary contribution

- Hydrogen Bonding: The magnetite component’s surface and potentially the MOF’s organic linkers can serve as either donors or acceptors of hydrogen bonds. The adsorbed state can be further stabilized by these groups forming hydrogen bonds with appropriate acceptor atoms in the dye molecule’s structure.
- Physical Adsorption (Physisorption) and Pore Filling:

The BET analysis indicated that the composite possesses a high surface area of 373.27 m²/g and features a mesoporous structure with an average pore size of approximately 4.75 nm. The pores offer a significant surface area and void spaces for the adsorption of dye molecules through non-specific van der Waals forces. The Dubinin-Radushkevich isotherm model yielded a mean adsorption energy (E) of 0.767 kJ/mol, significantly lower than 8 kJ/mol. This value indicates that the overall process is primarily governed by physisorption rather than chemisorption, thereby confirming the significance of this physical capture mechanism.

#### Mass transfer and kinetics

The kinetic study elucidates the rate-controlling step.

The adsorption equilibrium was attained within a brief period of 5 min. The process adhered to pseudo-second-order kinetics (R² = 1), indicating that the rate-limiting step likely involves an interaction between the adsorbent and adsorbate.

The intra-particle diffusion model indicated that the plot did not intersect the origin, suggesting that pore diffusion occurs but is not the only rate-limiting factor. Film diffusion, occurring through the boundary layer surrounding the particle, significantly influences the initial rapid adsorption phase.

##### So, the proposed mechanism can be illustrated as follows

- Initial mass transfer involves the diffusion of cationic Rhodochrome molecules through the solution to the surface of the Ni-MOF/Magnetite composite, characterized by film diffusion.
- The primary attachment occurs due to significant electrostatic attraction, whereby dye cations are attracted to the negatively charged surface of the adsorbent at pH levels exceeding 5.8.
- Molecular diffusion occurs within the mesopores of the material, a process referred to as pore diffusion. Upon entry, they are retained by:

Physisorption occurs through van der Waals forces within the pores.

Hydrogen bonding interactions with surface functional groups.

- Equilibrium is achieved swiftly when the rates of adsorption and desorption become equal, leading to a significant observed removal capacity.

### Regeneration

**Fig. 18:**
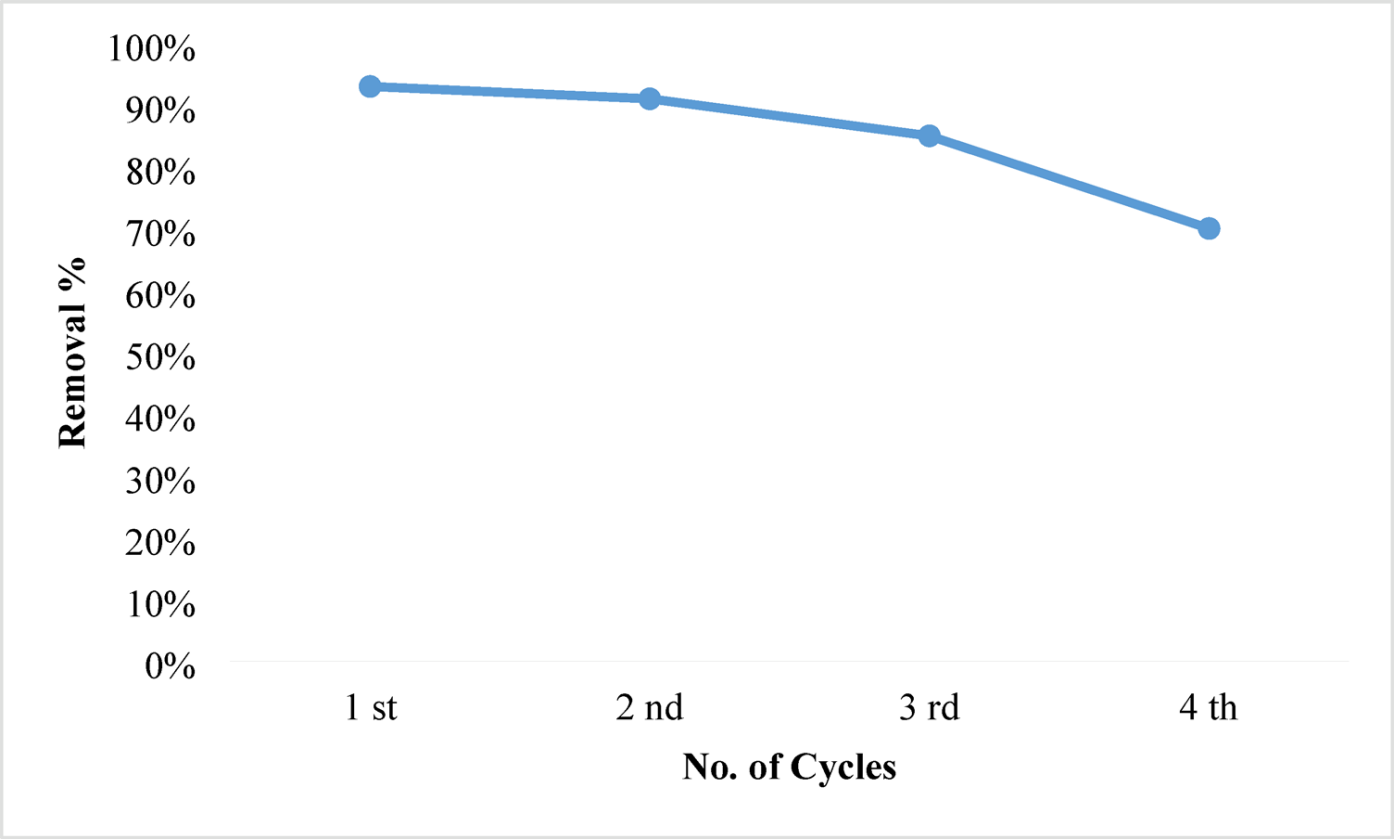
Number of cycles during the adsorption process.

Proving the eco-friendliness and economic feasibility of these NPs depends on their regeneration. The desorption phase is performed by rinsing the adsorbent with distilled water and then washing it with a solution of 0.1 mol/L of NaOH and HCl after the adsorption approach is finished. After three cycles of NPs, Fig. 18 revealed a decline in active site count and a removal efficiency of approximately 85%. The findings indicated they were reasonably priced material^108^.

## Economic Feasibility, environmental Impact, and industrial scalability

### Economic analysis and treatment costs

A preliminary economic analysis was conducted to evaluate the cost-effectiveness of using the synthesized Ni-MOF/Magnetite nanocomposite for Rhodochrome removal. The cost assessment considers the primary expenses: raw materials, synthesis (energy), and operational costs.

- Raw Material Costs: The synthesis utilizes nickel acetate, terephthalic acid, and iron salts, which are commercially available and relatively inexpensive. The use of green tea extract as a reducing and stabilizing agent for magnetite replaces more expensive and hazardous chemical agents (e.g., sodium borohydride), significantly reducing material costs and mitigating environmental hazards associated with synthesis.
- Synthesis Energy Costs: The synthesis protocols for both the Ni-MOF and the magnetite nanoparticles are performed at room temperature or near-ambient conditions (60 °C for drying). This low-energy requirement contrasts sharply with many other adsorbents that require high-temperature calcination or hydrothermal processes, leading to substantial energy savings.
- Operational Efficiency Costs: The operational costs are minimized due to the exceptional performance of the adsorbent:
  - High Efficiency: A 93% removal rate achieved within just 5 min drastically reduces the contact time required in a continuous flow system, leading to smaller reactor volumes and lower capital costs.
  - Low Dosage: The high efficiency is achieved with a very low adsorbent dosage (0.07 g/100 mL–0.7 g/L). This means less material is needed per liter of wastewater treated.
  - pH Flexibility: Effective operation at neutral to basic pH (7–9) means minimal need for costly pH adjustment acids or bases in many industrial effluents.
  - Reusability: The material maintained 85% efficiency after three regeneration cycles. This reusability spreads the initial synthesis cost over multiple treatment cycles, dramatically reducing the cost per liter of water treated.

A simplified cost comparison with conventional treatment methods (e.g., activated carbon, ion exchange resins) would show that while the nanomaterial itself might have a higher initial synthesis cost, its superior kinetics, capacity, and reusability make it highly competitive on a cost-per-volume-treated basis over its lifespan.

### Life-Cycle assessment (LCA)

A qualitative Life-Cycle Assessment (LCA) highlights the environmental merits of the proposed adsorbent system:

1. Resource Acquisition (Less Harmful): The utilization of non-toxic, plant-derived tea extract represents a renewable resource and is superior to synthetic, petroleum-derived reducing agents.
2. The environmentally friendly synthesis method for magnetite reduces the production of hazardous waste. The synthesis of Ni-MOF at room temperature adheres to green chemistry principles by minimizing energy requirements.
3. Operation (Highly Efficient): The rapid adsorption kinetics are directly associated with reduced energy consumption for mixing and pumping in a practical treatment plant.
4. End-of-Life and Regeneration: The magnetic properties of the nanocomposite represent a significant advantage. The adsorbent can be effortlessly extracted from the treated water utilizing an external magnet, so obviating the necessity for energy-consuming centrifugation or filtering. This additionally inhibits the emission of nanoparticles into the environment. The effective regeneration using mild acids or bases indicates that the adsorbed species can be condensed into a minimal volume of eluent for appropriate disposal or potential recovery, advancing towards a circular economy model instead of generating a substantial volume of contaminated sludge.

Possible Environmental Compromise: The primary environmental issue in the LCA concerns to the utilization of nickel, a potentially hazardous metal. This risk is reduced by the robust stability of the Ni-MOF structure and the effective magnetic recovery, which inhibits leaching and discharge into the aquatic ecosystem.

#### Green study

Also, the green assessment was conducted to evaluate the sustainability and environmental friendliness of the proposed analytical procedure for Rhodochrome removal. Two green metric methods were chosen: the Green Analytical Procedure Index (GAPI) and the Analytical Eco-Scale.

#### GAPI assessment

**Fig. 19:**
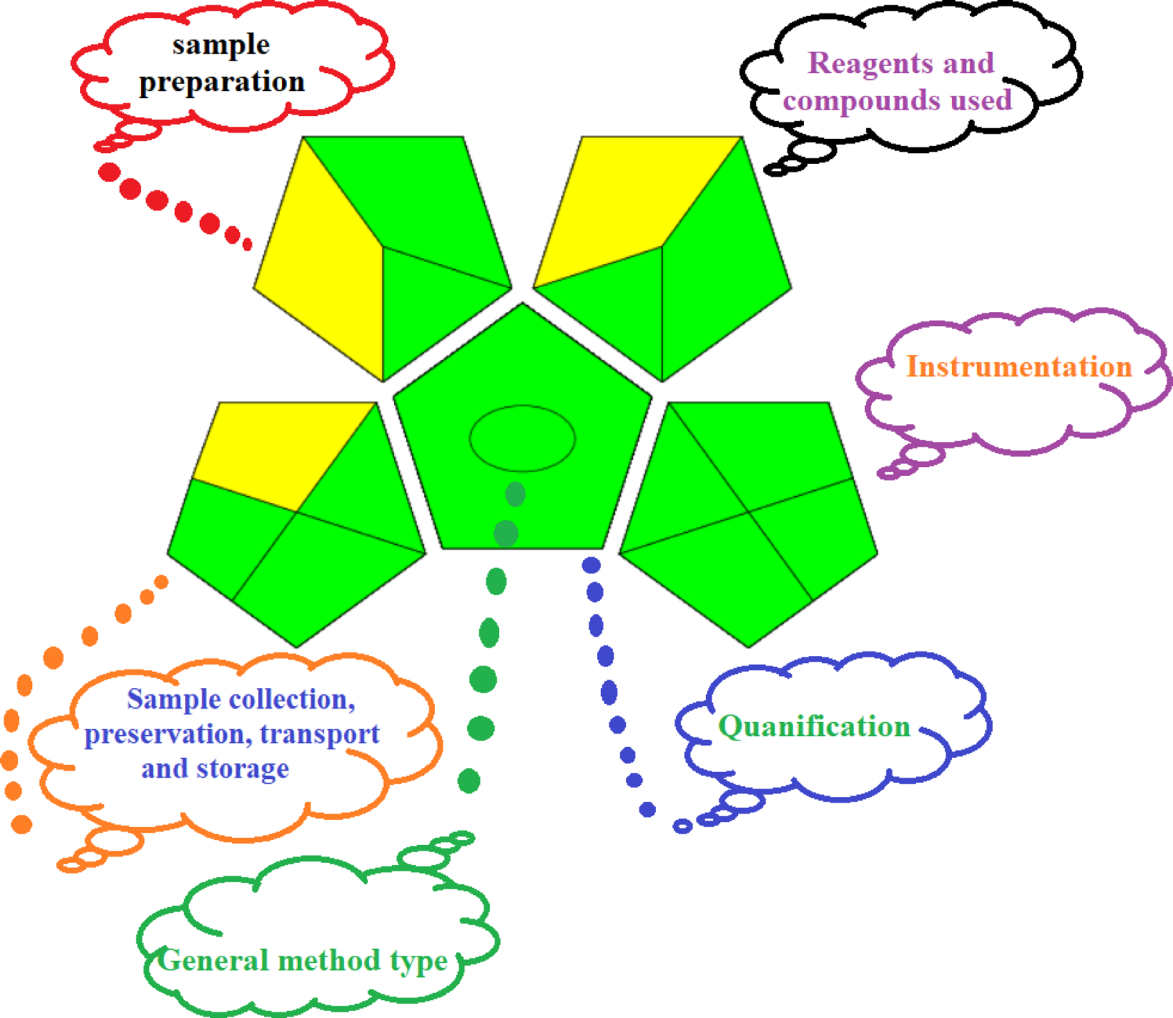
Green metric tool GAPI.

The Green Analytical Procedure Index (GAPI) is represented by pentagonal pictograms that employ red, yellow, and green colors, with green indicating a high level of greenness and red indicating the lowest level. The framework consists of five pentagrams that include sample preparation and analysis, sample collection and preservation, method type, solvents/reagents utilized, and instrumentation to classify a tested method as green or non-green. The proposed method is characterized by the synthesis of the adsorbent at room temperature using a green approach. It features efficient sample preparation, rapid analysis times, minimal waste generation, low energy consumption, and straightforward procedures without requiring additional steps or specialized sample storage. The proposed approach was validated as environmentally friendly, as demonstrated in Fig. 19, which displayed twelve green sections, three yellow sections, and no red Sects^109–111^.

#### The analytical Eco-Scale

The Analytical Eco-Scale is utilized to evaluate the environmental sustainability, eco-friendliness, and greenness of analytical methods. This method uses a penalty point system, in which points are subtracted from a maximum score of 100. The calculation of penalty points is based on the solvents or chemicals used energy consumption of instruments, occupational hazards, and waste generation. The Analytical Eco-Scale categorizes values above 75 as good and those above 50 as acceptable. If the value is below 50, it is classified as non-eco-friendly or non-green. Additionally, the quantity of hazard pictograms provided by the Globally Harmonised System of Classification and Labelling of Chemicals (GHS) and the corresponding safety data sheets for each chemical or solvent are considered. The Analytical Eco-Scale was employed to measure the overall greenness (see Table 7). The optimized analytical procedure for Rhodochrome removal attained an Eco-Scale score of 87, demonstrating that the method exceeds the threshold value of 75. This outcome demonstrates favorable environmental performance and underscores the method’s adherence to principles of green analytical chemistry^109–111^.

**Table 7:** Analytical Eco-scale method for the proposed method of evaluating environmental sustainability.

Hazard		Penalty points
Reagent	Deionized water	0
	NaOH	2
	Terephthalic acid (benzene-1,4-dicarboxylic acid)	2
	Nickel (II) acetate dihydrate	4
	Ferric chloride hexahydrate	2
	Iron(II) chloride tetrahydrate	2
Instruments	Energy < 0.1 KWh per sample	0
	Occupational waste (The procedure does not release vapors into the environment)	0
Waste	Amount of waste < 1 mL	1
	Management (no treatment)	0
Total penalty points	13	
Analytical Eco-Scale total score	87	

### Potential for Large-Scale industrial applications

The presented data strongly supports the potential for scaling up this technology for industrial applications:

- Technical scalability: The synthesis methods are simple, based on common precipitation and mixing techniques, and do not require sophisticated equipment. This makes them easily transferable from the lab to an industrial setting.
- Magnetic separation: This is the key to scalability. In an industrial continuous flow system, a magnetic separator can be integrated to continuously remove and recycle the adsorbent particles without clogging filters. This addresses the biggest challenge in using powdered adsorbents at a large scale.
- Target applications: This technology is particularly suited for:
  - Treatment of Industrial Dyeing and Printing Effluents: Specifically for waste streams containing cationic dyes.
  - Point-Source Treatment: It could be deployed for in-situ treatment at smaller workshops or factories that generate colored wastewater.

## Conclusion

Ni-MOF/Magnetite A green, eco-friendly, natural-dependent procedure was tested using SEM, XRD, TEM, BET, and zero-point charge methods before being used as a novel strategy to remove rhodochrome (kammererite) from wastewater using batch adsorption. At a dye concentration of 10 ppm and a pH of 8, 0.07 g of nanoparticles was utilized as an adsorbent. The removal rate was nearly 93% after five minutes at room temperature. The experimental findings demonstrated a significant correlation with a Temkin isotherm model, with a maximum regression coefficient of R^2^ of 0.9928 and calculated R^2^of 0.994. The maximum adsorption capacity, q_max_, was 13.58 mg/g. The kinetic data points to a pseudo-second order process that governs sorption. Regeneration studies have shown that NPs can be employed for three cycles with higher removal efficacy. Ni-MOF/Magnetite NPs enhanced rhodochrome dye removal from wastewater. So, this study encourages for the Ni-MOF/Magnetite nanocomposite as an effective, easily separable, and reusable adsorbent for the remediation of cationic dye pollution in wastewater, especially from textile and dyeing industries. The green synthesis protocol effectively substitutes toxic chemical agents with a plant-based alternative, presenting a safer and more sustainable approach for nanomaterial production. Additionally, it is essential to validate its performance in continuous-flow systems with actual industrial effluents to comprehensively evaluate its practical potential and economic viability.

The current study utilized food-grade materials for the synthesis and testing of nanomaterials. The positive results suggest that the extraction and synthesis method is viable and may be applicable to alternative sources. Future research should investigate the application of the same methodology to non-food agricultural waste as alternative precursors to enhance sustainability and decrease costs.

## Supplementary Information

Below is the link to the electronic supplementary material.

Supplementary Material 1

## Data Availability

All data generated or analyzed during this study are included in this published article.
