# Supplementary material for: Kinetic and isotherm study of Ni-MOF/Magnetite nanoparticles adsorption capacity as green synthesized adsorbent towards rhodochrome (Kammererite)
Source: Sci Rep. 2025 Dec 28;15:44669. doi: 10.1038/s41598-025-29707-7 (PMC12749104; doi:10.1038/s41598-025-29707-7)
Supplement: Supplementary file 1 — Supplementary Material 1 [file 41598_2025_29707_MOESM1_ESM.docx]

**Kinetic and Isotherm Study of Ni-MOF/Magnetite Nanoparticles Adsorption Capacity as Green Synthesized Adsorbent towards Rhodochrome (Kammererite)**

Mostafa F. Elshafei^1*^, Maysa R. Mostafa^1^, Perihan A. Khalf‐Alla^1^, Gehad G. Mohamed^12^, Omar A. Fouad^1*^

^1^ [Department of Chemistry, Faculty of Science, Cairo University, Giza, 12613, Egypt](https://www.benthamdirect.com/search?option1=pub_affiliation&value1=%22Department+of+Chemistry%2C+Faculty+of+Science%2C+Cairo+University%2C+Giza%2C+12613%2C+Egypt+%3B%22&option912=resultCategory&value912=ResearchPublicationContent).

^2^ [Department of Nanoscience, Faculty of Basic and Applied Sciences, Egypt-Japan University of Science and Technology, New Borg El Arab, Alexandria, 21934, Egypt](https://www.benthamdirect.com/search?option1=pub_affiliation&value1=%22Department+of+Nanoscience%2C+Basic+and+Applied+Sciences+Institute%2C+Egypt-Japan+University+of+Science+and+Technology%2C+New+Borg+El+Arab%2C+Alexandria%2C+21932%2C+Egypt%22&option912=resultCategory&value912=ResearchPublicationContent)


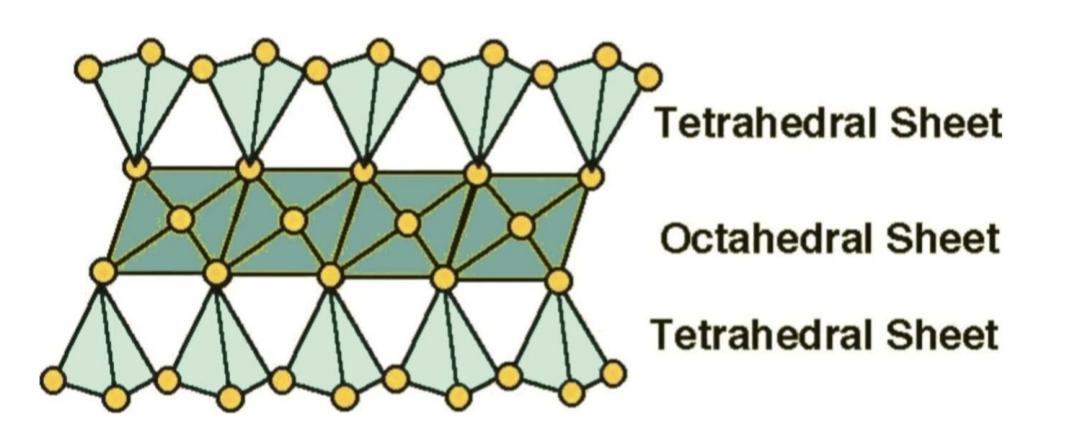


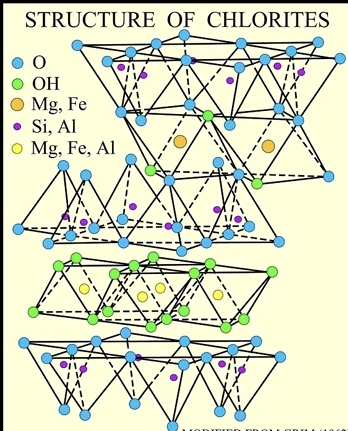
Supplementary figure 1. The structure of rhodochrome.
